# Divergent cytotoxic and inflammatory functions of intratumoral Vδ2^+^ γδ T cells in renal cell carcinoma

**DOI:** 10.3389/fimmu.2026.1864165

**Published:** 2026-07-17

**Authors:** Won Joon Oh, Jaeyoun Park, Tilanka Kalanapriya Dahanayake, Seon Yong Yeom, Heeju Ryu, Seong Il Seo, Tae Jin Kim

**Affiliations:** 1 Department of Immunology, Graduate School of Basic Medical Science, Sungkyunkwan University School of Medicine, Suwon, Republic of Korea; 2 Personalized Cancer Immunotherapy Research Center, Sungkyunkwan University Medical Research Center, Suwon, Republic of Korea; 3 Department of Urology, Samsung Medical Center, Sungkyunkwan University School of Medicine, Seoul, Republic of Korea

**Keywords:** CD16, flow cytometry, Renal cell carcinoma, scRNA analysis, spatial transcriptomics, Vδ^2^ γδ T cells, γδ T cell

## Abstract

Vγ9Vδ2 T cells are blood-circulating, innate-like lymphocytes that contribute to tumor immunosurveillance and constitute over 95% of Vδ2^+^ γδ T cells, but how they perform antitumor or protumor functions remains unclear. Here, we comprehensively characterized Vδ2^+^ γδ T cells in paired peripheral blood and tumor samples from patients with renal cell carcinoma using flow cytometry and single-cell transcriptomic analysis. We identified two major Vδ2^+^ γδ T cell subsets distinguished by expression of CD16, which were present at variable proportions in both circulation and tumors. Notably, the proportion of CD16^+^Vδ2^+^ γδ T cells in blood closely reflected those in matched tumor tissues. Functionally, CD16^+^Vδ2^+^ γδ T cells exhibited potent cytotoxic capacity, characterized by high granzyme B expression and enhanced degranulation upon activation. In contrast, CD16^–^Vδ2^+^ γδ T cells displayed central and effector memory features, expressed higher levels of CCR5, CXCR3, and granzyme K, and showed superior migratory capacity but limited cytotoxic activity. Single-cell RNA sequencing of tumor-infiltrating Vδ2^+^ γδ T cells identified four transcriptional clusters, including *FCGR3A*^+^ cytotoxic and *GZMK*^+^ inflammatory clusters corresponding to CD16^+^ and CD16^–^ subsets, respectively. A *TOX*^+^ cluster exhibited progenitor-like features, giving rise to two divergent effector states along a differentiation trajectory. Within tumors, both CD16^+^ and CD16^-^ γδ T cells were broadly distributed and CD16^+^ γδ T cells were more preferentially co-localized with tumor cells than CD16^-^ γδ T cells. Collectively, these findings demonstrate that blood and intratumoral Vδ2^+^ γδ T cells are functionally heterogeneous and segregate into cytotoxic and inflammatory effector programs.

## 1 Introduction

γδ T cells constitute a distinct arm of cellular immunity that integrates innate and adaptive immune responses. They recognize transformed or stressed cells through both innate receptors and γδ T cell receptors (γδ-TCRs) and are characterized by rapid cytokine release and MHC-unrestricted cytotoxicity without the need for specific MHC-restricted peptide priming (1–3). Their broad tissue distribution, stress-sensing capacity, and ability to mount effector responses independent of classical antigen presentation have positioned γδ T cells as promising candidates for next-generation cancer immunotherapies (2, 4). Consistent with this view, recent studies across human tumors revealed that γδ T cells assume diverse phenotypic and functional states, shaped by local inflammatory cues, metabolic conditions, and tumor-derived signals (5).

γδ T cells comprise both tissue-resident and blood-circulating populations (6), among which Vγ9^+^Vδ2^+^ T cells represent the predominant blood-circulating subset and respond sensitively to phosphoantigens generated from the dysregulated mevalonate pathway (7–9). Similar to other blood-circulating immune cells such as monocytes and conventional lymphocytes, Vγ9^+^Vδ2^+^ T cells migrate to sites of inflammation and exert effector functions upon entry into peripheral tissues (10, 11). Because both Vγ9^+^Vδ2^+^ T cells and tissue-resident Vδ1^+^ T cells are detected within human cancers (12, 13), the two γδ T cell populations are presumed to perform distinct antitumor or protumor roles within the tumor microenvironment (TME).

Despite their recognized role in tumor immunosurveillance (14), the mechanisms governing Vγ9^+^Vδ2^+^ T cell function in solid tumors remain incompletely understood. This ambiguity is compounded by the functional versatility of γδ T cells, which includes cytotoxicity, cytokine secretion, antigen presentation, and immune-regulatory activities (15, 16). In particular, it remains unclear whether cytotoxic Vγ9^+^Vδ2^+^ T cells represent a terminal differentiation state acquired upon tumor entry or whether cytotoxic and non-cytotoxic effector programs arise through distinct developmental pathways within the TME.

In this study, we examined Vδ2^+^ γδ T cells in paired blood and renal cell carcinoma (RCC) tumor samples to elucidate their phenotypic changes upon tumor entry and observed a functional division into CD16^+^ and CD16^-^Vδ2^+^ γδ T cell subsets prior to tumor infiltration. These subsets were phenotypically distinct, and single-cell RNA sequencing of RCC-infiltrating Vδ2^+^ γδ T cells revealed further heterogeneity, resolving for transcriptional clusters, one of which was identified as a progenitor-like population.

## 2 Materials and methods

### 2.1 Patients and clinical samples preparation

Blood and tumor samples were collected from treatment-naive ccRCC patients undergoing partial or radical nephrectomy at Samsung Medical Center (Seoul, Korea) between 2021 and 2023. The study was approved by the Institutional Review Board of Samsung Medical Center (IRB No: 2021-01-077) and written informed consent was obtained from all participants. **Supplementary Table 1** lists all the patients and their clinical information. Heparinized peripheral blood (PB) and tumor tissue specimens were collected intraoperatively. Tissue samples were obtained from non-necrotic and non-hemorrhagic central regions of the tumors.

Matched PB and tumor tissue samples were processed for lymphocyte isolation. Tumor tissues were mechanically dissociated into single-cell suspensions by mincing with surgical blades, followed by dilution in 20 ml wash buffer (PBS containing 4% FBS). Both blood and tumor cell suspensions were layered onto 15 ml Ficoll-Paque PLUS (Cytiva, Marlborough, MA, USA) in 50 ml conical tubes and centrifuged at 400 × g for 20 min at 20 °C with brake off. The lymphocyte-enriched interface was carefully collected using serological pipettes, transferred to fresh 50 ml tubes, and washed with 30 ml wash buffer by centrifugation at 300 × g for 10 min. Red blood cell contamination was eliminated by incubating cell pellets with 3 ml ACK Lysing Buffer (Thermo Fisher Scientific, Waltham, MA, USA) for 5 min at room temperature. Purified lymphocytes were washed twice with wash buffer and resuspended at 1 × 10^7^ cells/ml in cryopreservation medium (90% FBS, 10% DMSO). Cell viability was assessed by trypan blue exclusion and exceeded 90% for all samples.

### 2.2 Flow cytometry

Dead cells were excluded using Fixable Viability Dye eFluor 780 (eBioscience, San Diego, CA, USA) in PBS for 30 min at 4 °C prior to surface staining. Purified lymphocytes (0.5-1 × 10^6^ cells per sample) were then stained with fluorochrome-conjugated antibodies (**Supplementary Table 2**) in 100 μl FACS buffer (PBS containing 4% FBS and 0.1% sodium azide) for 30 min at 4 °C. Following surface staining, cells were washed twice with FACS buffer by centrifugation at 300 × g for 5 min.

For intracellular cytokine staining, surface-stained cells were fixed with Fixation Buffer (Invitrogen, Carlsbad, CA, USA; cat. no. 00-8222-49) for 30 min at room temperature. After fixation, cells were washed with 1× Permeabilization Buffer (diluted from 10× stock; Invitrogen, cat. no. 00-8333-56), then permeabilized and stained simultaneously using 1× Permeabilization Buffer containing intracellular antibodies for 30 min at room temperature. Cells were then washed twice with 1× Permeabilization Buffer, followed by a final wash with FACS buffer.

Flow cytometric acquisition was performed on a BD FACSCanto II flow cytometer (BD Biosciences) at the Biomedical Omics Research Platform (BIORP) of Korea Basic Science Institute (KBSI). Data were analyzed using FlowJo software version 10.10.0 (BD Biosciences, Franklin Lakes, NJ, USA). The gating strategy for total Vδ2^+^ γδ T cells and CD16^+^ or CD16^-^ Vδ2^+^ γδ T cells in PB and tumor samples are shown in **Supplementary Figure 1A**.

### 2.3 Migration assay

Transwell migration assays were performed using the Cell Migration/Chemotaxis Assay Kit (96-well, 8 μm) (Abcam, Cambridge, UK; cat. no. ab235673) according to the manufacturer’s instructions. CD16^+^ and CD16^-^ Vδ2 T cells were sorted using a BD FACSAria III cell sorter (BD Biosciences). Sorted cells (5 × 10^4^ cells in 100 μl RPMI) were seeded into upper chambers. Lower chambers were filled with 150 μl RPMI + FBS 10% (R10) containing recombinant human CCL5 (10, 50, or 100 ng/ml) or CXCL10 (10 or 100 ng/ml). As a negative control, R10 alone was added to the lower chamber.

After 4-hour incubation at 37 °C with 5% CO2, upper chambers were removed and discarded. Migrated cells in bottom chambers were centrifuged, washed twice with Wash Buffer, and incubated with Cell Dye and Cell Dissociation Solution for 60 min at 37 °C. Fluorescence (Ex/Em = 530/590 nm) was measured using a microplate reader, and migrated cell numbers were determined by interpolation from a standard curve generated with known cell numbers. All assays were performed in triplicate.

### 2.4 Cell stimulation and degranulation assay

For degranulation assays, 96-well flat-bottom plates were coated overnight at 4 °C with anti-CD3 antibody (clone OKT3; eBioscience, cat. no. 16-0037-81) at 2 or 5 μg/ml and anti-CD16 antibody (clone B73.1; eBioscience, cat. no. 16-0167-82) at 1 or 2 μg/ml in PBS. Plates were washed twice with PBS before use. Then, purified lymphocytes (3-5 × 10^5^ cells in 100 μl per well) were added to antibody-coated wells or stimulated with soluble phosphoantigens: 2 or 5 μg/ml isopentenyl pyrophosphate (IPP; Sigma-Aldrich, St. Louis, MO, USA; cat. no. 39784) or 0.5 or 1 μg/ml (E)-4-hydroxy-3-methyl-but-2-enyl pyrophosphate (HMBPP; Sigma-Aldrich, St. Louis, MO, USA; cat. no. 95098) in R10 medium supplemented with 20 μM TAPI-0 (Sigma-Aldrich, St. Louis, MO, USA; cat. no. SML1292). For detection of degranulation, CD107a antibody (clone H4A3; BioLegend, cat. no. 328616; 5 μl per well) was added directly to the culture at the start of the 4-hour stimulation, together with monensin (eBioscience, cat. no. 00-4505-51) and brefeldin A (eBioscience, cat. no. 00-4506-51), according to the manufacturer’s instructions, so that surface CD107a mobilized during degranulation was captured throughout the assay. After 4 hour incubation at 37 °C with 5% CO_2_, cells were washed with FACS buffer and stained for additional surface markers for 30 minutes at 4 °C, then washed, fixed, and analyzed by flow cytometry on a BD FACSCanto II (BD Biosciences). CD107a expression was quantified as a marker of degranulation and cytotoxic activity.

### 2.5 Processing and analysis of single-cell RNA-seq data

For single-cell analysis, γδ T and B cells were sorted from fresh tumor tissues of three ccRCC patients using a BD FACSAria III cell sorter (BD Biosciences). Sorted cells were immediately processed and submitted to Macrogen Inc. (Seoul, Korea) for library preparation and sequencing.

Single-cell libraries were prepared using the Chromium Controller (10x Genomics) according to the manufacturer’s protocol, targeting 10,000 cells per sample. Following barcoding, reverse transcription, and cDNA amplification in droplets, 5’ gene expression libraries were constructed. Libraries were quantified by qPCR (KAPA), quality-assessed using an Agilent 4200 TapeStation, and sequenced on a NovaSeq 6000 platform (Illumina).

Single-cell RNA sequencing (scRNA-seq) data were analyzed using Seurat package (version 5.3.1). Raw gene expression matrices were normalized via UMI counts, and low-quality cells characterized by high mitochondrial gene content were excluded. After quality control and cell type annotation, we retained a total of 2,846 Vδ2^+^ γδ T cells for the targeted single-cell transcriptome analysis. Following data scaling and initial principal component analysis (PCA), Harmony (version 1.2.4) was employed to integrate datasets from three individual patients, effectively mitigating inter-individual variability and batch effects. Unsupervised cell clustering was then performed using a graph-based approach based on the top 16 Harmony-corrected principal components with a resolution of 0.5. The resulting clusters were visualized using Uniform Manifold Approximation and Projection (UMAP), and major cell types were annotated based on the expression of lineage-specific marker gene.

For subtype identification, cells corresponding to each major cell type annotation were re-analyzed through iterative UMAP dimensionality reduction, graph-based clustering, and marker gene analysis. Differentially expressed genes among clusters were identified using the FindMarkers function in Seurat with the Wilcoxon rank-sum test, applying the following criteria: log fold change (logFC) > 0.25, adjusted p-value < 0.05, and minimum percentage of cells expressing the gene (min.pct) > 0.05.

### 2.6 T cell and NK cell state signature scores

To characterize the functional states of γδ T cell subpopulations, we computed state signature scores of each γδ T cell cluster based on canonical markers of T and NK cell differentiation status according to previous references. The signature genes for T and NK cells are listed below.

#### 2.6.1 T cell state gene signatures

Naive/Tcm: naive/central memory T cells; *CCR7, LEF1, SELL, TCF7, IL7R, CD27, CD28, BCL2* (17, 18).

Tem: effector memory T cells; *CCL5, GZMK, GNLY, EOMES, ZNF683, KLRG1, NKG7, ZEB2, CCL3, CCL4, CCL20, IFNG, IL10, TNF, XCL1, XCL2* (18).

Temra: terminally differentiated effector memory T cells; *KLRG1, PRF1, FGFBP2, CX_3_CR1, GZMB, GNLY, ZNF683, LAG3, CD160* (18).

Tex: exhausted T cells; *PDCD1, CTLA4, HAVCR2, TIGIT, TOX* (19).

TRM: tissue-resident memory T cells; *ITGA1, ITGAE, CD27, TUBA1A, VIM, LGALS1, LGALS3* (18, 20).

#### 2.6.2 NK cell state gene signatures

Immature NK: *NCAM1, CD27, IL7R, KLRC1, TCF7, XCL1, XCL2, AREG, SELL, GZMK* (21).

Transition NK: *GZMK, KLRC1, KLRG1, TIGIT, NCAM1* (21, 22).

Cytotoxic NK: *FCGR3A, CX_3_CR1, KLRB1, CD38, GZMB, PRF1, CD160, CD247, NKG7, FCER1G, CHST2, CLIC3, SPON2* (21, 22).

Adaptive NK: *B3GAT1, KLRC2, PRDM1, ZBTB38, CD2, IL32, CCL5, GZMH* (21–23).

TrNK: Tissue-resident NK cells; CD69, *ITGAE, ITGA1, CXCR6, ZNF683, IKZF3, AREG* (21).

#### 2.6.3 Four pathway scores

“T cell differentiation” (Gene Ontology Biological Process; GOBP_T_CELL_DIFFERENTIATION), “NK cell differentiation” (Gene Ontology Biological Process; GOBP_NK_CELL_DIFFERENTIATION), “T cell cytotoxic pathway” (BioCarta; BIOCARTA_TCYTOTOXIC_PATHWAY), and “NK cell−mediated cytotoxicity” (KEGG; KEGG_NATURAL_KILLER_CELL_MEDIATED_CYTOTOXICITY) (24).

Module scores for each signature were calculated using the AddModuleScore function in Seurat v5.3.1 (25) with default parameters. This method computes the average expression of genes within each signature relative to a control gene set binned by average expression levels, thereby accounting for technical variation in gene detection rates. For each cell, the module score represents the mean normalized expression of signature genes minus the mean expression of randomly selected control features.

For cluster-level comparative analysis, mean module scores were calculated by averaging individual cell scores within each cluster. To enable cross-signature comparison, these cluster-level scores were min-max normalized to a 0–1 scale: Normalized score = (score - min)/(max - min), where min and max represent the minimum and maximum scores across all clusters for each signature. Normalized scores were visualized using radar charts generated with the fmsb package in R to compare signature profiles across clusters. The spatial distribution of module scores was displayed on UMAP embeddings using FeaturePlot function in Seurat.

### 2.7 Trajectory analysis

To reconstruct the developmental trajectory and infer the differentiation dynamics of Vδ2^+^ T cells, we performed an integrated analysis using PAGA, Monocle 3, scVelo, and CellRank.

First, to infer the global topological connectivity and developmental hierarchy among the identified cell clusters, Partition-based Graph Abstraction (PAGA) was performed using Scanpy in Python (26). The PAGA graph provided a robust backbone revealing the branching architecture of the distinct functional lineages (27).

Following this, pseudotime trajectory analysis was performed using Monocle 3 (version 1.3.7) in R (28). Cells were ordered along a learned trajectory to infer differentiation pathways. The principal graph was constructed using the learn_graph function, and pseudotime values were assigned using the order_cells function with *TOX*+ cells designated as root nodes. Differential gene expression along pseudotime was identified using the graph_test function.

For RNA velocity analysis, we generated spliced and unspliced count matrices from the BAM files provided by Macrogen using Velocyto (version 0.17.17) with the refdata-gex-GRCh38-2020-A reference genome (29). During this process, we filtered out an additional 3 cells, resulting in a final dataset of 2,843 cells for the subsequent RNA velocity and trajectory inference analysis. We merged the resulting loom files were merged with the Seurat object and analyzed using scVelo (version 0.3.3) in Python (30). After filtering and normalizing the data, we employed the dynamical model (scv.tl.recover_dynamics) to estimate RNA velocities and calculate latent time, representing the cell’s internal clock derived from splicing dynamics.

To robustly predict cellular fate and visualize the directional flow of differentiation, we utilized CellRank (version 2.0.8) (31). We computed a transition matrix by combining two kernels to prioritize splicing kinetics while retaining the global directional trend. Specifically, we applied a weighted combination of 0.8 for the RNA velocity kernel (based on latent time) and 0.2 for the pseudotime kernel. A higher weight was assigned to the RNA velocity kernel to prioritize the transcriptional kinetics derived from the dynamical model, which captures the directional momentum of cellular differentiation. The pseudotime kernel (calculated via scVelo in deterministic mode, mode=‘deterministic’) was included with a lower weight to serve as a global constraint, ensuring the trajectory respects the continuous phenotypic progression from the progenitor-like *TOX*^+^ state to the terminally differentiated *FCGR3A*^+^ effector state, thereby mitigating local noise inherent in velocity vector fields. Differentiation streamlines were visualized on the UMAP embedding to delineate the dominant differentiation paths from the *TOX*^+^ progenitor-like cluster toward the differentiated effector states.

To rigorously quantify the developmental potential and infer the cellular hierarchy among the identified Vδ2^+^ T cell clusters, we utilized the CytoTRACE 2 computational framework. The CytoTRACE 2 algorithm predicts the developmental potential of a single cell by leveraging transcriptional complexity and the expression of genes associated with differentiation states, independent of prior knowledge of developmental pathways (32). The normalized gene expression matrix of Vδ2^+^ T cells was provided as input to calculate the continuous potency scores for individual cells. The resulting scores were then compared across the four clusters to evaluate their relative progenitor-like characteristics versus terminally differentiated states, confirming their identities within the mature lymphocyte spectrum.

### 2.8 Validation using a publicly available γδ T cell scRNA-seq dataset

To validate our findings in an independent cohort, we re-analyzed a publicly available scRNA-seq dataset of tumor-infiltrating γδ T cells from RCC (GEO: GSE223809; six patients pooled) (33). After standard quality control in Seurat (v5.3.1), Vδ2^+^ cells were isolated by *TRDV2* gating with exclusion of B cell (*CD79A/B*, *MS4A1*) and other Vδ chain (*TRDV1*, *TRDV3*) transcripts, yielding 788 cells that were clustered into four subsets (Louvain, resolution 0.2; UMAP). Differentiation potential was assessed with CytoTRACE 2 and transcriptional diversity, and a Monocle 3 pseudotime trajectory was rooted at the TOX-high cluster, as for our dataset (**Supplementary Figure 4**).

### 2.9 Gene set enrichment analysis

GSEA was performed using GSEA software (version 4.3.3, Broad Institute). C2 (KEGG pathway) and C5 (Gene Ontology) gene sets from the Molecular Signatures Database (MSigDB) were used as reference (24, 34). Normalized enrichment scores (NES) and false discovery rate (FDR) q-values were calculated. Gene sets with FDR q-value < 0.25 were considered significantly enriched.

### 2.10 Spatial transcriptomic analysis and neighborhood colocalization

Spot-level cell type assignment was performed using a module score–based annotation approach with predefined gene signatures: CD4+ T cells (*CD4, IL7R, CD3E, TRAC*), CD8+ T cells (*CD8A, CD3D, CD3E, TRAC, GZMB*), B cells (*MS4A1, CD79A, CD79B, CD19*), macrophages (*LYZ, C1QA, C1QB, C1QC, CD68, CD163*), NK cells (*NKG7, KLRD1, FCGR3A, GNLY*), and γδ T cells (*TRDC, TRGC1, TRGC2, TRGV9, XCL1*). Module scores were calculated using Seurat’s AddModuleScore function, and spots were assigned to the cell type with the highest module score using a winner-takes-all strategy, provided the maximum score exceeded a minimum threshold of 0.10. Spots below this threshold were classified as “Low Immune.” Tumor likelihood was estimated by logistic transformation of ccRCC module scores (*CA9, NDUFA4L2, VIM*). Spots with tumor likelihood > 0.90 were excluded from downstream immune analyses. CD16^+^ and CD16^−^ γδ T cell subsets were distinguished by *FCGR3A* expression within spots assigned to the γδ T cell category.

For spatial zone visualization, spots were clustered using the Louvain algorithm (35)(FindClusters; resolution = 0.08 and 0.4 for Patients #2 and #3, respectively) following principal component analysis. Zone-specific module scores were overlaid on tissue coordinates, and Voronoi tessellation (deldir package version 4) (36) was applied to delineate spatial boundaries between clusters, with edge length filtering and connected component analysis (igraph package) (37) to remove border artifacts and small fragments.

For spatial neighborhood analysis, each spot’s local neighborhood was defined as itself and its k = 6 nearest spatial neighbors, constructed using a custom k-nearest neighbor function reflecting the hexagonal geometry of the Visium array. Neighborhood composition was quantified by counting each immune cell type and summing tumor likelihood values within each neighborhood. Permutation-based enrichment testing (n = 1,000 iterations) was performed following the framework described by Palla et al. (38) to assess whether observed neighborhood compositions deviated significantly from spatial randomness. For each permutation, an equal number of center spots were randomly sampled from all eligible spots regardless of cell type. Enrichment Z-scores were calculated as Z = (μ_olm_ – μ_penm_)/σ_penm_, and two-sided empirical P-values were computed from the permutation distribution, with positive Z-scores indicating colocalization and negative Z-scores indicating spatial exclusion. Differential spatial positioning between CD16+ and CD16− γδ T cell subsets was assessed by comparing their neighborhood compositions using the Wilcoxon rank-sum test.

### 2.11 Statistical analysis

Statistical analyses were performed using GraphPad Prism (version 8.0), R (version 4.4.2), and Python (version 3.10.19). Flow cytometry comparisons between matched PBMCs and tumor-infiltrating lymphocytes from the same patient were performed using a paired Student’s t-test. For unpaired comparisons, an unpaired Student’s t-test or Mann-Whitney U test was applied as appropriate. Multiple group comparisons were analyzed by one-way ANOVA with Tukey’s *post-hoc* test. Correlation analyses were performed using Pearson or Spearman correlation coefficients. P-values < 0.05 were considered statistically significant. Data are presented as mean ± SD unless otherwise stated. Figures were generated using GraphPad Prism (version 8.0), R with the ggplot2 package (version 3.5.2), and Python (version 3.10.19).

## 3 Results

### 3.1 Cancer-infiltrating Vδ2^+^ γδ T cells show enhanced effector memory differentiation with increased tissue-residency markers

**Figure 1:**
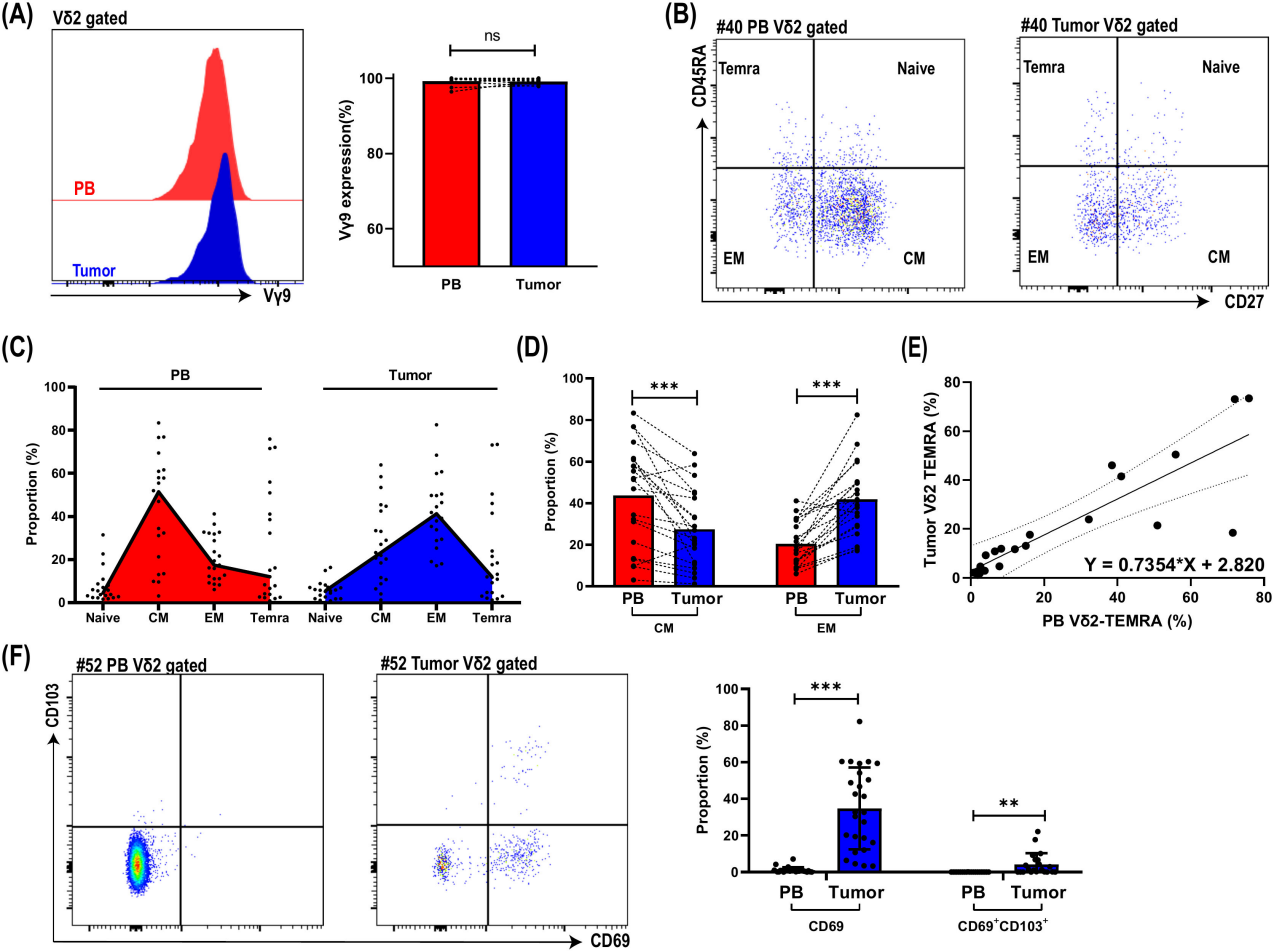
Distribution of blood and tumor-infiltrating Vδ2^+^ γδ T cell subpopulations based on CD45RA and CD27 and tissue residency markers CD69 and CD103. **(A)** Representative histogram of Vγ9 expression gated on Vδ2^+^ γδ T cells from PB (red) and tumor (blue) (left), and quantification of Vγ9^+^ frequency among Vδ2^+^ γδ T cells in PB versus tumor (right; *n* = 11 patients). ns, not significant. **(B–E)** Peripheral blood (PB, shown as red) and tumor-infiltrating (shown as blue) Vδ2^+^ γδ T cells were displayed for the expression of CD45RA and CD27 and the proportions of individual subpopulations were shown. Naïve, CD45RA^+^CD27^+^; CM (central memory), CD45RA^–^CD27^+^; EM (effector memory), CD45RA^–^CD27^–^; TEMRA (terminally differentiated effector memory re-expressing CD45RA), CD45RA^+^CD27^–^. **(B)** Gating strategy **(C)** Distribution of Vδ2^+^ γδ T cell subpopulations. Each dot represents a proportion of the given subpopulation among the total Vδ2^+^ T cells in an individual patient (*n* = 21 patients). **(D)** Comparison of proportions of CM and EM Vδ2^+^ γδ T cells in total Vδ2^+^ γδ T cells between matched PBMC and tumor-infiltrating Vδ2^+^ γδ T cells (*n* = 21 patients). Data are shown as mean ± SD. **(E)** Correlation analysis between TEMRA proportions in PBMC and tumor-infiltrating Vδ2^+^ γδ T cells from the same patients (*n* = 21 patients). Linear regression line with 95% confidence interval is shown. **(F)** Expression of CD69 and CD103 in PB (red) and tumor-infiltrating (blue) Vδ2^+^ γδ T cells. Representative flow cytometry plot of CD69 and CD103 expression on Vδ2^+^ T cells (left) and quantification of CD69^+^ and CD69^+^CD103^+^ tissue-resident Vδ2^+^ T cell proportions in PBMC versus tumor (right; *n* = 21 patients). Statistical significance was determined by paired t-test. **p < 0.01, ***p < 0.001.

To determine whether Vγ9^+^Vδ2^+^ T cells undergo phenotypic alterations upon tumor entry, we analyzed paired PB and tumor-infiltrating Vδ2^+^ γδ T cells from patients with clear cell RCC (ccRCC) using multiparametric flow cytometry. As previously reported, more than 98% of Vδ2^+^ γδ T cells were positive for Vγ9 both in PB and tumor (**Figure 1A**). Due to fluorochrome limitations in the multicolor panel, Vδ2^+^ γδ T cells were used for subsequent flow cytometric sorting and downstream analyses. In this study, we have primarily used the term “Vδ2^+^ γδ T cells” for the experimentally defined population throughout the manuscript, whereas the term “Vγ9^+^Vδ2^+^ T cells” is used when referring to the canonical phosphoantigen-responsive γδ T cell subset described in the literature.

Based on CD45RA and CD27 expression, we classified Vδ2^+^ γδ T cells into naive (CD45RA^+^CD27^+^), central memory (CM; CD45RA^–^CD27^+^), effector memory (EM; CD45RA^–^CD27^–^), and terminal effector memory re-expressing CD45RA (TEMRA; CD45RA^+^CD27^–^) subsets as shown for a representative gating for patient #40 (**Figure 1B**) (39, 40). The distribution of these subsets varied substantially among individual patients in both PB and tumor samples (**Figure 1C**). Despite this inter-individual variability, we observed consistent phenotypic differences between circulating and tumor-infiltrating Vδ2^+^ γδ T cells. Tumor tissues contained a significantly higher proportion of EM Vδ2^+^ γδ T cells than PB, suggesting enhanced activation and effector differentiation upon tumor entry (**Figure 1D**). In contrast, the proportion of CM Vδ2^+^ γδ T cells was significantly reduced in tumors relative to PB (**Figure 1D**). In parallel, tumor-infiltrating Vδ2^+^ γδ T cells expressed higher levels of the tissue-residency markers CD69 and CD103, as well as PD-1, and HLA-DR, than their circulating counterparts (**Figure 1F**; **Supplementary Figure 1B**).

Inter-individual variation in the proportion of TEMRA Vδ2^+^ γδ T cells was as pronounced as that observed for CM and EM subsets. However, the frequency of tumor-infiltrating TEMRA Vδ2^+^ γδ T cells positively correlated with that in matched PB (**Figure 1E**). Collectively, these data demonstrate that cancer-infiltrating Vδ2^+^ γδ T cells are enriched for effector memory populations and exhibit increased expression of tissue-residency markers, consistent with tumor-associated activation and differentiation within the TME, while the TEMRA Vδ2^+^ γδ T cell compartment appears to be independently regulated and characterized by marked inter-individual variability.

### 3.2 Variable numbers of two distinctive CD16^-^ and CD16^+^Vδ2^+^ γδ T cells are present both in blood and cancer tissues

**Figure 2:**
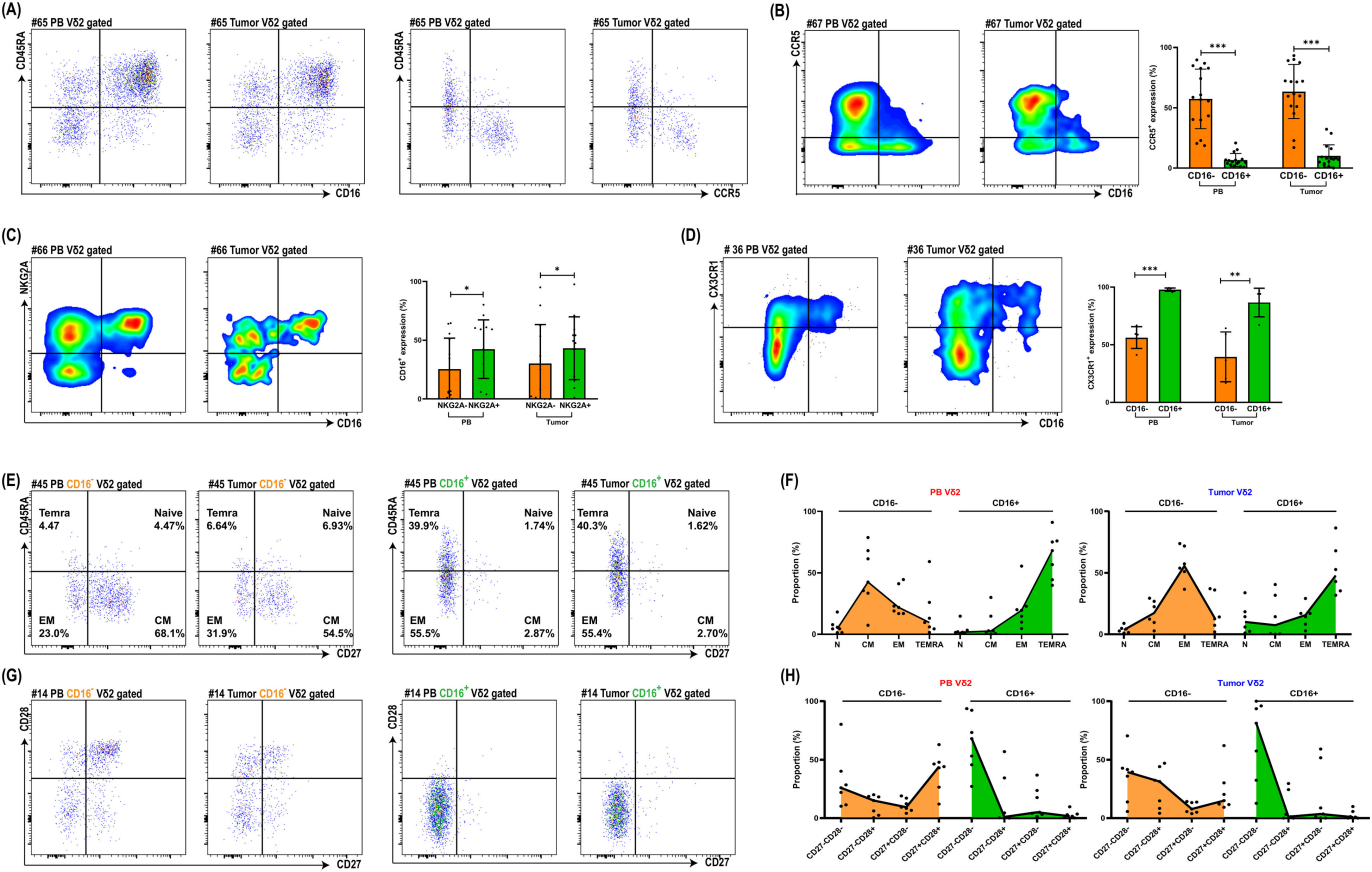
Division of Vδ2^+^ γδ T cells based on the CD16 expression. **(A)** Representative flow cytometry plots showing the expression of CD45RA and CD16 (left) or CCR5 (right) on Vδ2^+^ γδ T cells from PB and tumor. **(B, D)** Representative flow cytometry plot showing the expression of CD16 and CCR5 **(B)** or CX_3_CR1 **(D)** on Vδ2^+^ γδ T cells (left). Quantification of the percentages of CCR5^+^ **(B)** or CX_3_CR1^+^ **(D)** cells in CD16^–^ (orange) and CD16^+^ (green) Vδ2^+^ γδ T cells from PB and tumor (right; CCR5, *n* = 16 patients; CX_3_CR1, *n* = 5 patients). **C** Representative flow cytometry plot showing the expression of CD16 and NKG2A on Vδ2^+^ γδ T cells (left). Quantification of the percentages of CD16^+^ cells in NKG2A^–^ (orange) and NKG2A^+^ (green) Vδ2^+^ γδ T cells from PB and tumor (right; PBMC, *n* = 19 patients; Tumor, *n* = 16 patients). **(E, F)** Representative flow cytometry plot showing the expression of CD27 and CD45RA on CD16^–^ (orange) and CD16^+^ (green) Vδ2^+^ γδ T cells **(E)**. Distribution of naïve **(N)**, central memory (CM), effector memory (EM), and TEMRA subsets within CD16^–^ or CD16^+^ Vδ2^+^ γδ T cells from PB (left; *n* = 7 patients) and tumor (right; *n* = 7 patients) **(F)**. **G-H** Representative flow cytometry plot showing the expression of CD27 and CD28 on CD16^–^ (orange) and CD16^+^ (green) Vδ2^+^ γδ T cells **(G)**. Distribution of T cell subpopulations based on the CD27 and CD28 expression within CD16^–^ or CD16^+^ Vδ2^+^ γδ T cells from PB (left; *n* = 7 patients) and tumor (right; *n* = 7 patients) **(H)**. Statistical significance was determined by paired t-test. *p < 0.05, **p < 0.01, ***p < 0.001.

Since TEMRA Vδ2^+^ γδ T cells appeared to be a subpopulation independent of other Vδ2^+^ γδ T cell subpopulations, we investigated the differential immunologic profiles of CD45RA^+^ and CD45RA^-^ Vδ2^+^ γδ T cells. CD45RA^+^Vδ2^+^ γδ T cells expressed higher levels of CD16 and lower levels of CCR5 than CD45RA^–^ cells (**Figure 2A**) The proportions of CD45RA^+^CD16^+^ vs CD45RA^-^CD16^-^ Vδ2^+^ γδ T cells were highly variable in different patients, but their proportions in tumor tissues were correlated well with those in blood samples from the same patients (**Figure 1E**; **Supplementary Figure 1G**).

We next investigated the correlation of the proportions of CD16^+^ Vδ2^+^ γδ T cells with sex, age, stage or grade of ccRCC patients. Female patients tended to have a higher proportion of CD16^+^ Vδ2^+^ γδ T cells than male patients (**Supplementary Figure 1C**). there was a trend toward increased frequencies of CD16^+^ Vδ2^+^ γδ T cells with advancing age in both PB and tumor tissues; however, this association did not reach statistical significance (**Supplementary Figure 1D**). Although no significant association was observed between tumor stage or grade and the frequency of CD16^+^ or CD16^–^ Vδ2^+^ γδ T cell subsets, the overall frequency of tumor-infiltrating Vδ2^+^ γδ T cells tends to decrease with increasing tumor grade (**Supplementary Figure 1E**).

Notably, the CD16 expression was largely mutually exclusive with the CCR5 expression within the Vδ2^+^ γδ T cell compartment (**Figure 2B**). Accordingly, three major subpopulations—CCR5^+^CD16^–^, CCR5^–^CD16^+^, and CCR5^–^CD16^–^—could be delineated based on CD16 and CCR5 expression (**Figure 2B**). The CD16 expression was also mutually exclusive with the CXCR3 expression (**Supplementary Figure 1F**). Importantly, expression of CCR5 and CXCR3 was more strongly associated with CD16 expression than with tissue location, indicating that these phenotypic characteristics are intrinsic to Vδ2^+^ γδ T cell subsets rather than solely shaped by tissue residency.

Next we investigated the relationship between the expression of CD16 and NKG-2A since previous studies described NKG2A^+^ and NKG2A^–^ Vδ2^+^ γδ T cell subsets having differential effector functions (41). We found that most CD16^+^Vδ2^+^ γδ T cells expressed NKG-2A (**Figure 2C**). Both NKG2A^+^ and NKG2A^–^ Vδ2^+^ γδ T cell subsets infiltrated tumors to comparable extents. Since CX_3_CR1 acts as a chemokine receptor for cytotoxic effector CD8^+^ T cells (42, 43), we measured its expression in two Vδ2^+^ γδ T cell populations. CD16^+^Vδ2^+^ γδ T cells expressed higher CX_3_CR1 levels than CD16^-^ Vδ2^+^ γδ T cells, suggesting that CD16^+^ and CD16^-^ Vδ2^+^ γδ T cells utilize distinct chemokine receptor programs to migrate into the tumor microenvironment (TME) and are differentially regulated by NK cell inhibitory molecule NKG2A (**Figure 2D**).

Furthermore, CD16^+^ and CD16^–^ Vδ2^+^ γδ T cells also exhibited clear phenotypic differences based on CD45RA and CD27 expression (**Figures 2E, F**). CD16^+^ Vδ2^+^ γδ T cells were preferentially enriched within TEMRA and EM compartments, whereas CD16^–^ Vδ2^+^ γδ T cells were predominantly distributed within CM and EM compartments—preferentially within CM in PB and within EM in tumor tissues (**Figures 2E, F**). The classification based on expression of CD27 and CD28 further confirmed that CD16^+^ Vδ2^+^ γδ T cells correspond to fully differentiated CD27^–^CD28^–^ TEMRA cells, whereas CD16^–^ Vδ2^+^ γδ T cells comprised CM (CD27^+^CD28^+^) and multiple EM populations (CD27^+^CD28^–^, CD27^–^CD28^+^, and CD27^–^CD28^–^) (**Figures 2G, H**). Together, these data indicate that human Vδ2^+^ γδ T cells comprise phenotypically distinct CD16^+^ and CD16^–^ subsets that coexist in circulation and cancer tissues but differ markedly in their differentiation status and adaptation to the TME.

### 3.3 Single-cell transcriptomic analysis of cancer-infiltrating Vδ2^+^ γδ T cells reveals four distinct clusters

**Figure 3:**
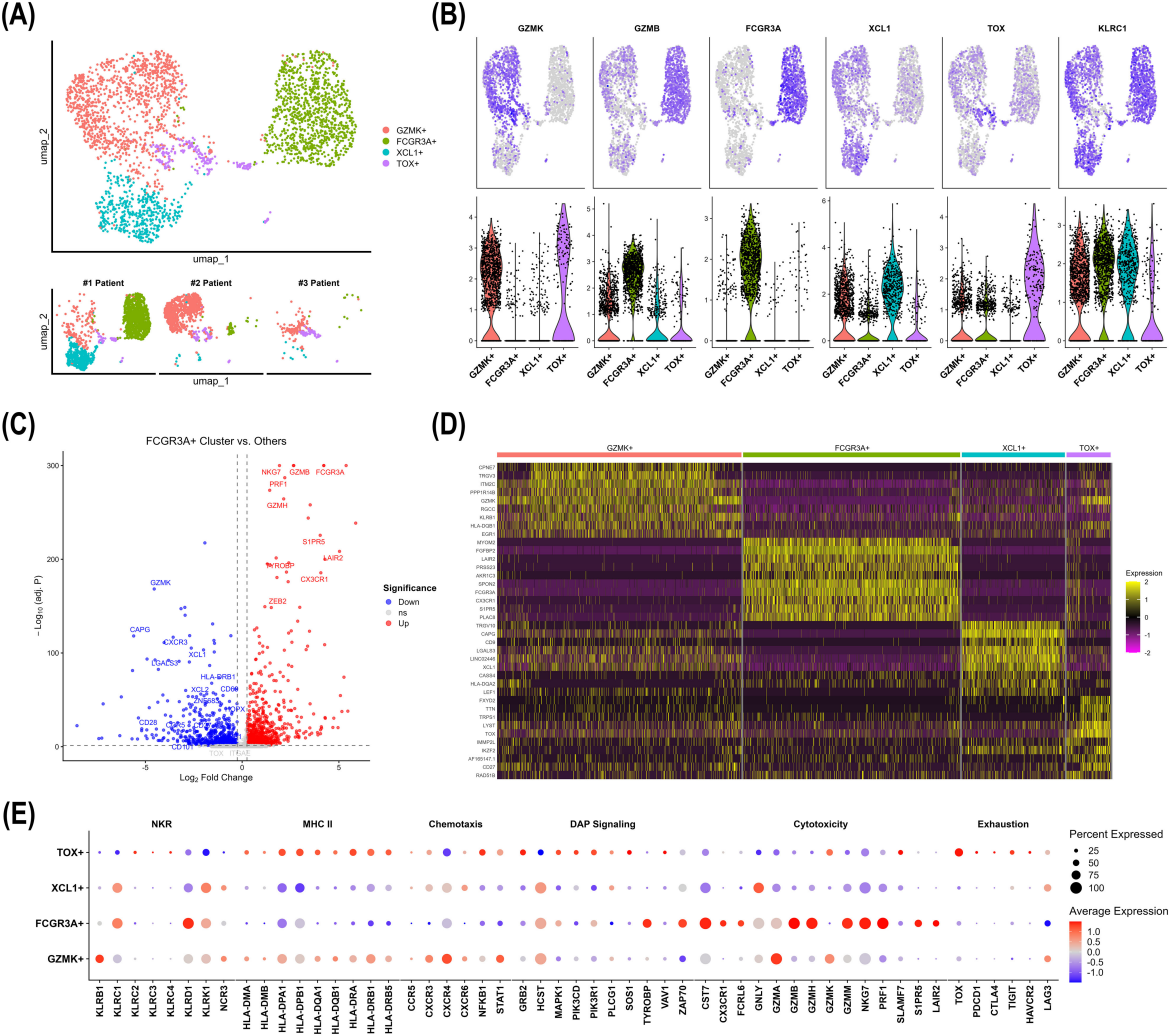
Four distinct transcriptional clusters of tumor-infiltrating Vδ2^+^ γδ T cells identified by single-cell RNA sequencing and UMAP dimensionality reduction. **A)** UMAP visualization showing the landscape of cell clusters from integrated data of tumor-infiltrating Vδ2^+^ γδ T cells from three patients. The upper panel displays four major clusters identified as *GZMK^+^* (n = 1,143), *FCGR3A^+^* (n = 1,015), *XCL1^+^* (n = 482), and *TOX^+^* (n = 206) clusters. The lower panel illustrates distributions of cells derived from three individual patients (#1, #2 and #3). **(B)** Expression of key marker genes - *GZMK, GZMB, FCGR3A, XCL1, TOX* and *KLRC1*- in four clusters. Feature plots showing the indicated gene expression profile projected on UMAP (upper panel). Violin plots comparing the expression levels of indicated markers across the identified clusters (lower panel). **(C)** Volcano plot showing DEGs between the *FCGR3A*^+^ cluster and the other clusters. Red, upregulated; blue, downregulated (Log_2_FC > 0.25, adj. p < 0.05); gray, not significant. Selected gene names are indicated. **(D)** Heatmap displaying the scaled expression of differentially expressed genes specific to indicated clusters. Scaled expression levels are color-coded (yellow, high; blue, low). **(E)** Dot plot analysis of functional gene modules; NK receptors (NKR), MHC class II, Chemotaxis, DAP signaling, Cytotoxicity, and Exhaustion. The dot size represents the percentage of cells expressing the indicated gene in given clusters, and the average expression levels are shown as color-coded scales (red, high; blue, low).

To precisely define the cellular heterogeneity of Vδ2^+^ γδ T cells within the TME, we performed single-cell RNA sequencing of sorted Vδ2^+^ γδ T cells from freshly resected RCC specimens of three patients. We performed the R package “harmony” to remove batch effects of samples (44) (**Supplementary Figure 2**). Following uniform manifold approximation and projection (UMAP) dimensionality reduction, clustering analysis revealed four transcriptionally discrete clusters, each characterized by a unique gene expression profile (**Figure 3A**). Based on their dominant gene signatures, we annotated as these clusters as *GZMK^+^* (n = 1,143), *FCGR3A^+^* (n = 1,015), *XCL1^+^* (n = 482), and *TOX^+^* (n = 206) clusters (**Figure 3B**). The *TOX*^+^ cluster expressed at levels comparable to *GZMK*, which lacked *TOX* expression. Notably, the distribution of four clusters varied drastically among the patients: patient #1 favored *FCGR3A*^+^ and *XCL1*^+^ clusters, patient #2 favored the *GZMK*^+^ cluster, and patient #3 had a dominant *TOX*^+^ cluster (**Figures 3A** bottom).

Since the *FCGR3A*^+^ cluster corresponds to the CD16^+^Vδ2^+^ γδ T cells that were identified by flow cytometry, we compared its gene expression profile against the other three clusters using the volcano plot (**Figure 3C**). The *FCGR3A*^+^ cluster expressed higher levels of *CX_3_CR1* and *KLRC1* (NKG-2A) and lower levels of *CD27, CD28, CCR5*, and *CD69* than the other three clusters, confirming it represents CD16^+^Vδ2^+^ γδ T cells. Canonical marker genes and the top 10 differentially expressed genes (DEGs) highlighted distinct functional programs for each of the four clusters (**Figure 3D**). We interpreted that CCR5^+^CD16^-^ Vδ2^+^ γδ T cells comprised the *GZMK*^+^, *XCL1*^+^, and *TOX*^+^ clusters as shown in functional module analysis using a dot blot (**Figure 3E**).

The *FCGR3A*^+^ cluster highly expressed typical cytotoxicity genes such as *GZMB*, *GZMH*, *PRF1*, and *NKG7* along with DAP signaling modules, essential for cytotoxic effector function (**Figure 3E**). Notably, this cluster also expressed *S1PR5* and *LAIR2*, which blocks the inhibitory interaction between LAIR-1 and collagen. Meanwhile, the *GZMK*^+^ cluster expressed *GZMK, GZMA, KLRB1* (CD161, NKR-P1A)*, CXCR4*, *STAT1*, and MHC class II genes (**Figures 3C, E**). We interpreted *TOX*^+^ cluster as a chronically activated T cells expressing exhaustion-related (*PDCD1, CTLA4*, and *TIGIT*) and MHC class II genes. The *XCL1*^+^ cluster distinctively expressed *KLRK1* (NKG-2D) and *GNLY* and appears to represent a different type of killer cells (45). Notably, the four clusters expressed distinct chemokine receptors, suggesting differential recruitment into the TME. Beyond the differential expression of CCR5, CXCR3, and CX_3_CR1 in CD16^+^ and CD16^-^ subsets, the four clusters exhibited even more distinct chemokine receptor gene expression profiles, such as *CXCR4* by the *GZMK*^+^ cluster and *CXCR2* by the *FCGR3A*^+^cluster. Collectively, these single-cell data demonstrate that intratumoral Vδ2^+^ γδ T cells are heterogeneous and segregate into functionally four distinct clusters.

### 3.4 Integrated genetic analyses reveal functional specialization of *FCGR3A*^+^, *GZMK*^+^, and *XCL1*^+^ clusters and progenitor-like potential of *TOX*^+^ cluster

**Figure 4:**
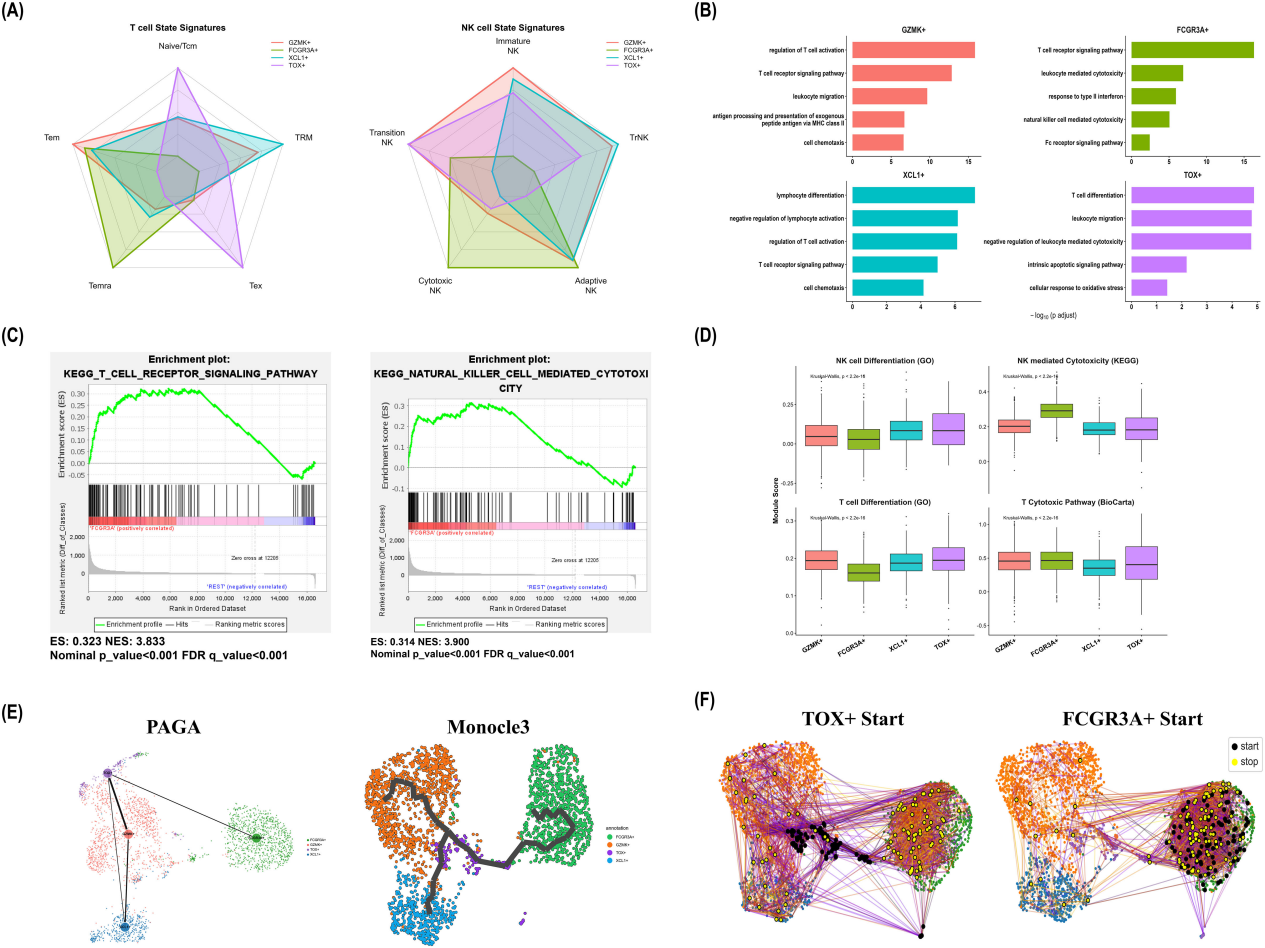
Functional characterization and developmental trajectory of Vδ2^+^ γδ T cell clusters. **A** Projection of established T cell and NK cell gene signatures onto the four Vδ2^+^ γδ T cell clusters. **B** Gene Ontology (GO) biological process enrichment analysis for individual clusters. Selected pathways that are significantly enriched in each cluster (FDR < 0.05) are shown with -log10(Padj) values. **C** GSEA plots for *FCGR3A*+ cluster showing enrichment of KEGG pathways: “T cell receptor signaling pathway” (left) and “Natural killer cell mediated cytotoxicity” (right). Normalized enrichment scores (NES) and FDR q-values are indicated. **D** Boxplots showing the module scores for T cell differentiation (GO), NK cell differentiation (GO), T cytotoxic pathways (Biocarta), and NK mediated cytotoxicity (KEGG) across the four clusters. Scores were calculated using the AddmoduleScore function in Seurat based on predefined gene sets. **E** Trajectory inference analyses using PAGA and Monocle3. PAGA connectivity graph (left) shows relationships between clusters with edge thickness representing connectivity strength. Monocle3 pseudotime trajectory (right) with cells colored by cluster identity. **F** CellRank-directed differentiation analysis utilizing scVelo-derived dynamics. The streamline plots depict the differentiation flow and endpoints when black colored cells from the *TOX*^+^ (left) or the *FCGR3A*^+^ clusters (right) are defined as developmental starting points. All analyses were performed on integrated single-cell RNA-seq data from n = 3 patients (same dataset as **Figure 3**).

To elucidate the functional states and developmental hierarchy of the four Vδ2^+^ γδ T cell clusters, we performed an integrated analysis combining gene signature projection, functional enrichment, and trajectory inference (**Figure 4**). Because Vδ2^+^ γδ T cells share features of both CD8^+^ T cells and NK cells (46), we projected established T cell and NK cell state signatures onto each cluster to define their phenotypic identities (**Figure 4A**). The *FCGR3A*^+^ cluster exhibited strong enrichment for TEMRA T cell and cytotoxic NK cell signatures. Consistent with these signatures, this cluster expressed high levels of canonical cytotoxic genes, including *GZMB*, supporting its identity as a terminally differentiated effector population with enhanced cytotoxic potential in both T cell NK cell maturation frameworks.

In contrast, based on both T cell and NK cell signatures, the *GZMK*^+^ cluster closely resembled those of the *XCL1*^+^ cluster (**Figure 4A**). Both clusters were enriched for EM and tissue-resident memory (Trm) signatures, suggesting that they represent activated effector populations with tissue adaptation features and potential antigen-presenting capacity via MHC class II. Within this group, the *GZMK*^+^ cluster expressed *GZMK* and *GZMA*, consistent with an inflammatory effector phenotype. The *XCL1*^+^ cluster expressed cytotoxicity-associated genes such as *GNLY* and *KLRK1* (NKG-2D), indicating a distinct killing potential. Notably, the *TOX*^+^ cluster enriched for naïve/CM and exhaustion-associated (Tex) gene signatures, suggesting a state of adaptation to chronic stimulation with progenitor-like features.

In functional enrichment analysis, the *FCGR3A*^+^ cluster significantly enriched for direct killing pathways, such as “Leukocyte mediated cytotoxicity” and “NK cell-mediated cytotoxicity”, validating its role as a terminal cytotoxic effector (**Figure 4B**). Consistently, the gene set enrichment analysis (GSEA) confirmed that the *FCGR3A*^+^ cluster strongly associated with TCR signaling pathway (NES = 3.83, FDR q < 0.001) and NK cell-mediated cytotoxicity pathways (NES = 3.90, FDR q < 0.001) (**Figure 4C**). Meanwhile, the *GZMK*^+^ cluster showed enrichment for “Regulation of T cell activation,” “Leukocyte migration,” and “Antigen processing and presentation” (**Figure 4B**). These results indicate that *GZMK*^+^ cells function primarily as inflammatory cells that likely orchestrate broader immune responses by recruiting immune cells to the TME and activating other immune cells, rather than serving as frontline killers. Comparing module scores revealed that *FCGR3A*^+^ cluster had a lower T cell differentiation score and a higher NK cell-mediated cytotoxicity pathway score than the other three clusters (**Figure 4D**).

To understand the potential developmental trajectories of the four clusters, we applied PAGA (Partition-based Graph Abstraction) and Monocle 3, an unsupervised inference method (**Figure 4E**). Both analyses consistently identified the *TOX*^+^ cluster as the phenotypic root of the hierarchy. The PAGA graph revealed a branching architecture where the *TOX*^+^ cluster gave rise to differentiated *GZMK*^+^ and F*CGR3A*^+^ lineages. Monocle 3 pseudotime analysis independently confirmed this hierarchical structure, placing the *TOX*^+^ cluster at the trajectory root. scVelo-derived RNA velocity streamlines confirmed a directional transcriptional flow originating from the *TOX*^+^ cluster (**Supplementary Figures 3A–C**), and latent time analysis further placed the *FCGR3A*^+^ cluster at the most advanced stage along this trajectory (**Supplementary Figure 3C**). To quantify their developmental potential, we assessed potency scores using CytoTRACE2. This scoring revealed that the *TOX*^+^ cluster exhibited the highest developmental potential among the four subsets, whereas the *FCGR3A*^+^ cluster displayed the lowest (**Supplementary Figure 3D**). Although all four Vδ2^+^ clusters fell within the “differentiated” range of potency scores, confirming their identity as mature lymphocytes rather than pluripotent stem cells. The *TOX*^+^ cluster displayed significantly higher developmental potential compared to the other clusters, particularly the *FCGR3A*^+^ cluster. Furthermore, tracking gene expression along this latent time trajectory revealed a progressive transition of key lineage-driving genes, clearly delineating the temporal shift from the *TOX*^+^ progenitor-like state to the F*CGR3A*^+^ effector state (**Supplementary Figures 3E–G**).

Furthermore, we explored the directional dynamics of these cells using RNA velocity and CellRank analyses (**Figure 4F**). RNA velocity vector fields illustrate the predicted future states of individual cells based on splicing kinetics, delineating the differentiation flow by simulating trajectories with directional streamlines. When we set the *TOX*^+^ cluster (which possesses the highest differentiation potential) as the starting point, the streamlines traverse through intermediate states and predominantly terminate at the *FCGR3A*^+^ cluster. Conversely, when the *FCGR3A*^+^ cluster is designated as a starting point, the streamlines exhibit negligible outward flow and remain predominantly within the *FCGR3A*^+^ population. Collectively, these results support a unidirectional differentiation model where *TOX*^+^ cells serve as a local progenitor-like reservoir that diverges into distinct functional lineages, ultimately giving rise to the terminally differentiated, NK-like cytotoxic *FCGR3A*^+^ population.

To confirm that this differentiation hierarchy was not specific to our cohort, we re-analyzed an independent public scRNA-seq dataset of tumor-infiltrating Vδ2^+^ γδ T cells from RCC (GSE223809). Consistent with our findings, the *TOX*^+^ cluster again showed the highest developmental potential by CytoTRACE 2 and was placed at the root of the Monocle 3 trajectory, while cytotoxic effector genes (GZMB, GZMK, and FCGR3A) were up-regulated relative to the TOX^+^ root along the trajectory (**Supplementary Figure 4**).

### 3.5 CD16^+^ are more closely localized to cancer cells than CD16^-^ γδ T cells

**Figure 5:**
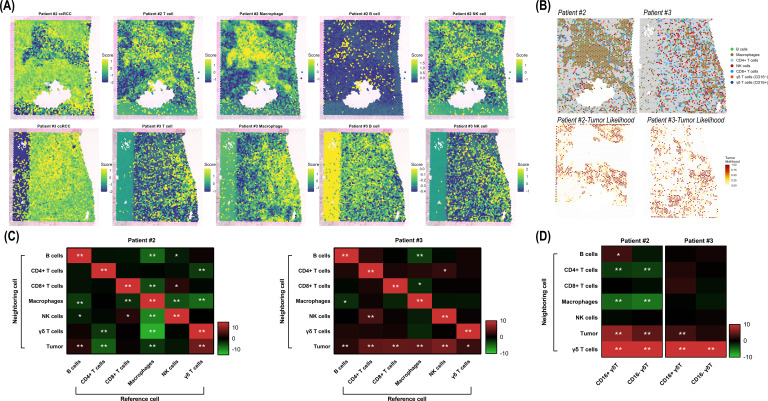
Spatial transcriptomics analysis of tumor microenvironment architectures showing CD16^+^ and CD16^-^ γδ T cells, other immune cells, and renal cell carcinoma cells. **(A)** 10x Visium spatial transcriptomics analysis showing module scores for indicated cell type signatures in ccRCC specimens from patients #2 (top row) and #3 (bottom row). Heatmaps display the densities of ccRCCs, T cells, macrophages, B cells, and NK cells according to given gene signature scores overlaid on H&E-stained tissue sections. Color intensity represents module score values (yellow, high; blue, low). Scale bars indicate score intensity. **(B)** Spatial mapping of individual cell types identified by deconvolution analysis (top panels). Spots are colored by predicted dominant cell type: B cells (green), macrophages (brown), CD4^+^ T cells (light blue), CD8^+^ T cells (sky blue), NK cells (dark red), CD16^+^ γδ T cells (orange), and CD16^-^ γδ T cells (navy). Bottom panels show tumor likelihood scores with high-tumor regions (red, high; yellow, intermediate) excluded from immune analyses. **(C)** Cell-cell neighborhood colocalization analysis using permutation-based enrichment testing (n=1,000 permutations). Heatmaps show colocalization Z-scores between reference cell types (rows) and neighboring cell types (columns) for patient #2 (left) and patient #3 (right). Red indicates significant positive colocalization; green indicates spatial exclusion. **p < 0.01, *p < 0.05. **(D)** Comparative neighborhood analysis of *FCGR3A*^+^ (CD16^+^) versus *FCGR3A*^-^ (CD16^-^) γδ T cell spatial positioning. Heatmaps show mean neighborhood composition for CD16^+^ and CD16^-^ γδ T cell spots in both tumor specimens. Color intensity represents enrichment scores for neighboring cell types. **p < 0.01, *p < 0.05.

To compare the spatial distribution of CD16^+^ and CD16^–^ γδ T cells within tumor tissues, we performed 10x Visium spatial transcriptomic analysis on tumor specimens from two ccRCC patients, #2 and #3 (**Figure 5**). Due to resolution problem, we could not discriminate between Vδ2^+^ and Vδ2^-^ γδ T cells. Using Squidpy module score analysis of predefined ccRCC, T cell, macrophage, B cell, and NK cell gene signatures, spatial mapping revealed markedly distinct TME architectures between the two specimens (**Figure 5A**). In tumor #2, ccRCC-enriched regions segregated spatially from immune cell–enriched regions, with immune signatures predominantly localized to stromal areas peripheral to tumor nests. In contrast, tumor #3 exhibited extensively intermingled tumor and immune cell signatures with T cell, macrophage, or B cell modules. Based on these spatial patterns, we classified tumor #2 and #3 as immune-excluded and immune-infiltrated phenotypes, respectively. Both CD16^+^ and CD16^–^ γδ T cells were scattered throughout both tumor and stromal regions in both cases (**Figure 5B**). Therefore, γδ T cells did not clearly cluster within the immune cell–enriched regions.

To precisely quantify the extent of immune cell infiltration into the tumor, we performed neighborhood colocalization analysis using permutation-based enrichment testing (n = 1,000 permutations). We visualized the colocalization scores between reference and neighboring cells as heat maps (**Figures 5C, D**). The number of immune cells neighboring γδ T cells or random spots showed that macrophages were more abundant than tumor cells in patient #2 but less abundant in patient #3 (**Supplementary Figure 5**). In both cases CD16^+^ γδ T cells are closer to tumor cells than CD16^-^ γδ T cells. Upon neighboring colocalization computation, γδ T cell spots were significantly colocalized with tumor spots in both tumor specimens (Z = +8.06, *p* < 0.01 for #2; Z = +2.76, *p* < 0.05 for #3). In contrast, B cell, CD4^+^ T cell, CD8^+^ T cell, macrophage, and NK cell spots did not colocalize with tumor cell or γδ T cell spots in the tumor #2 but did so in tumor #3 (**Figure 5C**). In the tumor #3, tumor cell spots also colocalized with all the other tested immune cell spots. Notably, macrophage spots spatially excluded other immune cell spots in both tumors, suggesting distinct macrophage-dominated niches exist. These data quantitatively confirm that γδ T cells closely contact tumor cells in both immune-excluded and immune-infiltrated tumors.

We next examined whether CD16^+^ and CD16^−^ γδ T cell subsets, defined by the *FCGR3A* expression, exhibited differential spatial positioning relative to tumor cells (**Figure 5D**). Direct comparison of neighborhood composition revealed that CD16*^+^* γδ T cell spots were surrounded by significantly more tumor spots than CD16*^-^* γδ T cell spots in both tumor specimens (mean 2.37 vs 1.66, *p* < 0.05 for #2; mean 1.75 vs 1.12, *p* < 0.05 for #3). The CD16*^+^* γδ T cell spots colocalized with more B cell spots in tumor #2 (mean 0.28 vs 0.07, *p* < 0.05) and more CD8^+^ T cell spots in tumor #3 (mean 0.71 vs 0.35, *p* < 0.01) than CD16*^-^* γδ T cell spots, suggesting differential interaction of CD16^+^ and CD16^−^ γδ T cells with other immune cells in different contexts. Collectively, these spatial analyses validate the single-cell transcriptomic findings and demonstrate that both CD16^+^ and CD16^−^ γδ T cells infiltrate into the tumor parenchyma. Furthermore, CD16^+^ γδ T cells preferentially infiltrate tumor-proximal regions, positioning them as potential mediators of direct antitumor cytotoxicity. A limitation of this spatial analysis is that multicellular Visium spots, with CD16^+^ and CD16^–^ γδ T cells defined by FCGR3A alone, could not discriminate between Vδ1^+^ versus Vδ2^+^ cells. Because CD16 expression has been reported on Vδ1^+^ γδ T cells (1) and Vδ1^+^ cells dominated the RCC γδ T cell infiltrate (12, 33), the tumor-proximal CD16^+^ γδ T cells identified here may include more Vδ1^+^ cells than Vδ2^+^ cells.

### 3.6 CD16^+^ Vδ2^+^ T cells exhibit stronger cytotoxic activity than CD16^–^ Vδ2^+^ T cells

**Figure 6:**
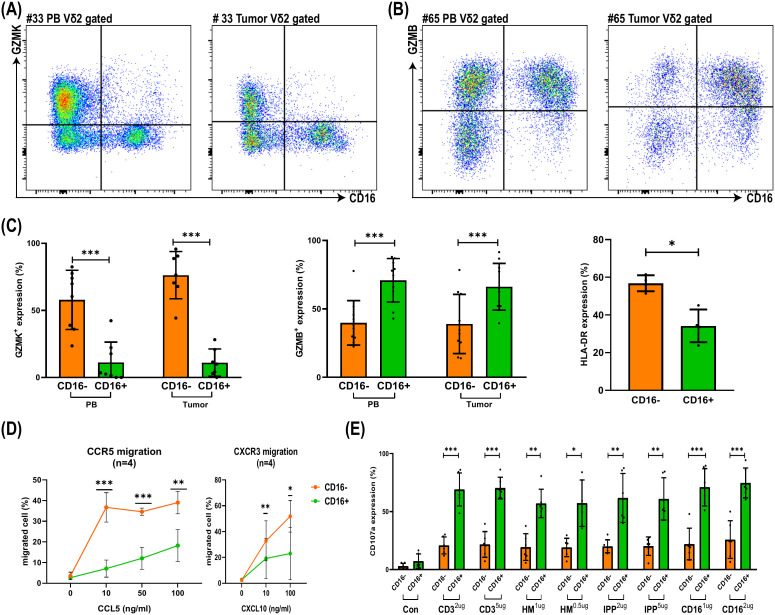
CD16^+^Vδ2^+^ γδ T cells exhibit high cytotoxic potential and distinct migratory responses compared to CD16^-^Vδ2^+^ γδ T cells. **A–B** Representative flow cytometry contour plots displaying for the expression of granzyme K (GZMK) **(A)** and granzyme B (GZMB) **(B)** with CD16 in PB-derived **(left)** and tumor-infiltrating (right) Vδ2+ γδ T cells from representative patient. **(C)** Quantitative comparison of the expression of granzyme K (GZMK), granzyme B (GZMB), and HLA-DR between CD16^–^ (orange) and CD16^+^ (green) Vδ2^+^ γδ T cells from PB and tumor samples. Bar graphs show frequencies of GZMK^+^ (left; *n* = 8 patients), GZMB^+^ (middle; *n* = 10 patients), and HLA-DR^+^ (right; *n* = 4 patients) cells within CD16^–^ and CD16^+^ Vδ2^+^ γδ T cell subsets. Each data point represents an individual patient; bars show mean ± SD. Statistical significance was determined by paired t-test. **(D)** Transwell migration assays showing chemotactic responses to CCL5 (left) and CXCL10 (right). Dose-response curves demonstrate differential migration capacity of CD16^–^ (orange) and CD16^+^ (green) Vδ2^+^ γδ T cells from peripheral blood. Data represent mean ± SD from four patients (*n* = 4 patients), each assayed in technical triplicate. **(E)** CD107a degranulation assay following 4-hour stimulation with various stimuli. CD16^-^ (orange) and CD16^+^ (green) Vδ2^+^ γδ T cells were stimulated with control medium (Con), anti-CD3 (2 or 5 μg/ml), HMBPP (0.5 or 1 μg/ml), IPP (2 or 5 μg/ml), or anti-CD16 (1 or 2 μg/ml) antibodies. The CD107a degranulation assay was performed on five independent patients blood samples (*n* = 5 patients). Data are presented as mean ± SD. Statistical significance was determined by paired t-test for matched samples. *p < 0.05, **p < 0.01, ***p < 0.001.

To confirm functional differences between CD16^+^ and CD16^–^ Vδ2^+^ γδ T cells, we examined granzyme K and B expression in PB and tumor-infiltrating Vδ2^+^ γδ T cells (**Figures 6A, B**). In three representative cases, CD16^+^ Vδ2^+^ γδ T cells rarely expressed granzyme K but frequently expressed granzyme B. In contrast, more than half of CD16^–^ Vδ2^+^ γδ T cells expressed granzyme K, whereas approximately 40% of these cells expressed granzyme B. These expression differences between the two populations were statistically significant (**Figure 6C**). CD16^–^Vδ2^+^ γδ T cells also expressed higher levels of HLA-DR compared with CD16^+^Vδ2^+^ γδ T cells, further supporting their inflammatory and antigen-presenting features. We evaluated their distinct migratory properties using transwell migration assays with CCL5 and CXCL10 (**Figure 6D**). Consistent with the higher expression of CCR5 and CXCR3, CD16^–^Vδ2^+^ γδ T cells migrated more efficiently than CD16^+^Vδ2^+^ γδ T cells.

In contrast, CD16^+^Vδ2^+^ γδ T cells exhibited stronger cytotoxic activity than CD16^–^Vδ2^+^ γδ T cells (**Figure 6E**; **Supplementary Figure 6**). Upon stimulation with solid-phase anti-CD3 or anti-CD16 antibodies, or with soluble phosphoantigens HMBPP or IPP, CD16^+^Vδ2^+^ γδ T cells upregulated CD107a to a greater extent than CD16^–^Vδ2^+^ γδ T cells. In these assays, the ADAM17 inhibitor TAPI-0 was included in the culture to prevent activation-induced shedding of CD16. Collectively, these results demonstrate that CD16^+^ and CD16^–^ Vδ2^+^ γδ T cells exhibit distinct functional properties, characterized by differential granzyme expression profiles, migratory capacities, and cytotoxic activities.

## 4 Discussion

Despite their substantial potential as MHC-independent cellular therapeutics, the clinical efficacy of Vγ9Vδ2 T cell-based immunotherapies remains limited. A major obstacle to maximizing their therapeutic impact is our incomplete understanding of how these cells phenotypically and functionally adapt upon entering the complex TME. To address this critical knowledge gap, we mapped the high-resolution transcriptional, functional, and spatial landscape of tumor-infiltrating Vδ2^+^ γδ T cells in ccRCC.

Our study demonstrates that intratumoral Vδ2 γδ T cells are composed of four distinct subpopulations by single-cell transcriptome analysis, and that two of the four clusters can be readily distinguished by flow cytometry based on the expression of CD16 and CCR5. By integrating multiparametric flow cytometry, single-cell transcriptomics, and trajectory inference, we demonstrate that these phenotypically defined subsets correspond to divergent functional programs within the TME. CD16^+^ Vδ2^+^ γδ T cells exhibit features of terminally differentiated cytotoxic effectors, characterized by TEMRA-like differentiation, high expression of cytotoxic molecules, and enhanced degranulation capacity (47). In contrast, CCR5^+^CD16^–^ Vδ2^+^ γδ T cells display an inflammatory effector program marked by increased migratory capacity, expression of inflammatory effector genes, and limited cytotoxic activity. Furthermore, trajectory analyses position the *TOX*^+^ cluster upstream of both cytotoxic and inflammatory effector states, suggesting that intratumoral Vδ2^+^ γδ T cells undergo functional diversification rather than linear maturation. Together, our findings provide a model in which circulating Vδ2^+^ γδ T cells infiltrate tumors and diverge into inflammatory, cytotoxic, and chronic activation programs, probably shaped by local microenvironmental cues that are individually varied.

The divergent differentiation observed in intratumoral Vδ2^+^ γδ T cells mirrors key features of CD8^+^ αβ T cell differentiation under chronic antigenic stimulation (48). In CD8^+^ T cells, sustained antigen stimulation can give rise to distinct functional states including cytotoxic terminal effector cells, inflammatory effector populations, and progenitor-like exhausted cells that maintain proliferative capacity (49–52). Our trajectory analysis suggests that a similar organizational principle may apply to γδ T cells in tumors. The *TOX*^+^ cluster exhibited transcriptional features associated with chronic activation and exhaustion-related programs, yet retained higher developmental potential compared with the other clusters. We interpreted that the *TOX*^+^ cluster contains both progenitor-like and terminally exhausted populations since TOX was shown to be expressed from precursors of exhausted T cells (53). From this progenitor-like state, Vδ2^+^ γδ T cells appeared to diverge toward a cytotoxic *FCGR3A*^+^ lineage and an inflammatory *GZMK*^+^ lineage. Such functional branching resembles the differentiation architecture reported for tumor-infiltrating CD8^+^ T cells and suggests that γδ T cells, despite their innate-like characteristics, may undergo analogous adaptive differentiation processes within the tumor microenvironment.

Another notable observation in this study is that the proportions of CD16^+^ and CD16^–^ Vδ2^+^ γδ T cells varied substantially among patients, yet the proportions observed in PB were correlated with those in tumor tissues from the same individuals. This finding suggests that the composition of tumor-infiltrating CD16^+^ and CD16^–^ Vδ2^+^ γδ T cells may be inferred from circulating Vδ2^+^ γδ T cell populations, potentially allowing estimation of intratumoral γδ T cell states without invasive tumor sampling. The basis for the functional heterogeneity among Vδ2^+^ γδ T cells remains unclear. One possibility is that this heterogeneity is established before tumor development through genetic background or prior environmental exposures that shape the circulating Vγ9Vδ2 T cell compartment. Alternatively, the heterogeneity may arise from ongoing antitumor or tumor-associated immune responses that dynamically influence Vδ2^+^ γδ T cell differentiation. In this scenario, tumor-reactive Vδ2^+^ γδ T cells may recirculate between tumor tissues and the systemic circulation, thereby contributing to the observed correlation between blood and tumor populations (54, 55). These mechanisms are not mutually exclusive, and both may operate to varying degrees in different individuals. Consistent with this possibility, variability in the proportion of CD16^+^ Vδ2^+^ γδ T cells has also been reported in healthy individuals in previous studies as well as in our own analysis of healthy donor samples (56). Collectively, the correlation between blood and tumor proportions supports a model in which pre-existing CD16^+^ cytotoxic and CD16^–^ inflammatory Vδ2^+^ γδ T cell subsets enter tumors with distinct differentiation states and subsequently undergo further adaptation within the TME.

This model also suggests that intratumoral Vδ2^+^ γδ T cells may divide labor between direct tumor cytotoxicity and orchestration of local immune responses. Such functional specialization could allow γδ T cells to participate both in direct tumor elimination and in shaping broader antitumor immunity. CD16^+^ Vδ2^+^ γδ T cells preferentially expressed granzyme B and exhibited robust degranulation responses, consistent with their classification as terminal cytotoxic effector cells. In contrast, CD16^–^ Vδ2^+^ γδ T cells displayed higher expression of granzyme K and HLA-DR, features associated with inflammatory effector functions and potential antigen-presenting capacity. Granzyme K-dominant cytotoxic programs have been described in inflammatory T cell subsets that contribute more to cytokine production and immune regulation than to direct target-cell killing (57, 58). This function of CD16^–^ inflammatory Vδ2^+^ γδ T cells appears to stimulate other immune cells such as dendritic cells, CD4^+^ and CD8^+^ T cells persistently in the TME.

The distinct chemokine receptor profiles observed in Vδ2^+^ γδ T cell subsets further support functional specialization in their recruitment and positioning within tumors. CD16^–^ Vδ2^+^ γδ T cells preferentially expressed CCR5 and CXCR3, receptors commonly associated with migration toward inflammatory chemokines such as CCL5 and CXCL10. In contrast, CD16^+^ Vδ2^+^ γδ T cells expressed higher levels of CX_3_CR1, a chemokine receptor frequently associated with terminal cytotoxic effector CD8^+^ T cells and NK cells. These findings suggest that cytotoxic and inflammatory Vδ2^+^ γδ T cells may be recruited to tumors through partially distinct chemotactic programs. Spatial transcriptomic analysis further supported this interpretation by demonstrating preferential localization of CD16^+^ γδ T cells in tumor-proximal regions, consistent with their role as direct tumor-killing effectors. Differential chemokine receptor expression may therefore represent an important mechanism governing the spatial organization and functional division of γδ T cell subsets within tumors.

In contrast to conventional αβ CD8^+^ T cells, the phenotypic changes observed in Vδ2^+^ γδ T cells after entry into tumor tissues were relatively modest, although an increased expression of tissue-residency markers such as CD69 and CD103 was detected in tumor-infiltrating Vδ2^+^ γδ T cells. We also observed an increased EM/CM ratio within the CD16^–^ Vδ2^+^ γδ T cell population, whereas most CD16^+^ Vδ2^+^ γδ T cells already exhibited a TEMRA phenotype. This limited phenotypic shift likely reflects the intrinsic activation status of circulating Vδ2^+^ γδ T cells. Even the CM-like Vδ2^+^ γδ T cells expressed CCR5 rather than CCR7, suggesting that they are already primed for migration to peripheral inflammatory sites. CCR5^+^ Vδ2^+^ γδ T cells therefore appear poised to rapidly enter inflamed tissues, including tumors, where they may contribute to the maintenance of an immunostimulatory microenvironment (59). Consistent with this interpretation, CCR5^+^Vδ2^+^ γδ T cells have been reported to be depleted in patients with acquired immunodeficiency, highlighting their role in inflammatory immune responses (60). Taken together, these observations support the idea that CD16^+^ and CD16^–^ Vδ2^+^ γδ T cells represent two pre-existing effector populations that are already primed to respond to inflammatory cues or infection and subsequently adapt to the tumor microenvironment after infiltration.

TCR clonotype analysis can provide valuable evidence regarding the developmental relationships inferred by the trajectory analyses. In particular, if circulating Vγ9Vδ2 T cells enter tumors and subsequently diversify into distinct functional states, clonally related cells would be expected to be shared among the inferred populations. Due to technical limitation, we could not investigate whether the *TOX*^+^, *FCGR3A*^+^, and *GZMK*^+^ subpopulations share clonotypes at the single-cell level. Nevertheless, our previous TCR repertoire studies provide independent evidence supporting a relationship between circulating and tumor-infiltrating Vγ9^+^Vδ2^+^ T cells. In glioblastoma, we reported preferential enrichment of Vγ9-Jγ2 rearrangements within tumors compared with blood (61). We recently published a similar analysis in ccRCC patients and observed enrichment of Vγ9-Jγ2 clonotypes in tumor tissues together with the presence of shared clonotypes between blood and tumor samples (62). These findings suggest that circulating Vγ9^+^Vδ2^+^ T cells can infiltrate tumors and undergo selective expansion or differentiation within TME.

The metabolic and transcriptional landscape of ccRCC may provide additional cues for the recruitment and activation of Vγ9Vδ2 T cells. ccRCC is characterized by constitutive activation of hypoxia-inducible factor (HIF) signaling due to frequent loss of *VHL* or equivalent mutations (63), resulting in a pseudohypoxic transcriptional program and oncogenic signaling in collaboration with additional renal lineage–specific factors (64). Sustained HIF-2α activation has been shown to promote lipid accumulation, including cholesterol and neutral lipid deposition, in ccRCC cells through metabolic reprogramming and activation of sterol regulatory element-binding protein-dependent pathways (65–68). Because cholesterol biosynthesis proceeds through the mevalonate pathway, which generates phosphoantigen intermediates such as isopentenyl pyrophosphate (IPP), dysregulated sterol metabolism in ccRCC may be associated with altered intracellular levels of these metabolites (69). Elevated phosphoantigen availability has been shown to activate Vγ9Vδ2 T cells in other tumor contexts, raising the possibility that the pseudohypoxic and lipogenic state of ccRCC could provide metabolic cues for Vγ9Vδ2 T cell recognition.

In addition to metabolic reprogramming, HIF-2α signaling contributes to an immunologically active phenotype in ccRCC, including modulation of antigen presentation pathways and interferon-responsive gene expression, which are associated with increased CD8^+^ T-cell infiltration in subsets of tumors (70). Hypoxia-driven chemokine production, including CCL5 and CX_3_CL1, has also been reported in various tumor contexts and can promote recruitment of CCR5^+^ and CX_3_CR1^+^ immune cell populations (71, 72). Such chemotactic gradients may facilitate infiltration of CCR5^+^ and CX_3_CR1^+^ Vγ9Vδ2 T cells into the TME. Thus, the pseudohypoxic program of ccRCC may simultaneously generate metabolic cues capable of activating Vγ9Vδ2 T cells and chemokine signals that guide their recruitment. Once within the TME, additional local factors—such as chronic antigenic stimulation, inflammatory cytokines, and cellular interactions—are likely to shape whether infiltrating Vγ9Vδ2 T cells adopt cytotoxic, inflammatory, or chronic activation states. Together, this framework provides a plausible mechanistic link between the unique metabolic landscape of ccRCC and the functional diversification of intratumoral γδ T cells observed in our study.

While further studies using larger patient cohorts and *in vivo* lineage tracing are needed to validate these trajectories and determine if this diversification extends beyond ccRCC, our findings and further analysis of previously published data (33) clearly demonstrate that tumor-infiltrating Vδ2^+^ γδ T cells undergo profound functional adaptation within the TME. Identifying a TOX^+^ progenitor-like state that gives rise to terminally differentiated CD16^+^ effector cells provides a new perspective on γδ T-cell biology in cancer. These insights may inform the design of future γδ T cell–based immunotherapies. By selectively isolating, expanding, or engineering specific subsets, such as the cytotoxic CD16^+^ population, or by modulating the TME to promote favorable differentiation trajectories, it may be possible to improve the efficacy of MHC-independent cellular therapies for solid tumors.

Because CD16 (FcγRIII) is a major mediator of antibody-dependent cellular cytotoxicity (ADCC), CD16^+^ Vδ2^+^ γδ T cells may cooperate with therapeutic monoclonal antibodies and thereby augment anti-tumor responses. In the context of ccRCC, it will be of particular interest to determine whether these cells can synergize with antibody-based therapies targeting VEGF/VEGFR pathways or other tumor-associated antigens (73). Moreover, the close correlation between the frequencies of CD16^+^ Vδ2^+^ γδ T cells in PB and tumor tissues raises the possibility that circulating CD16^+^ Vδ2^+^ γδ T cells could serve as a biomarker for identifying patients most likely to benefit from γδ T cell–directed or antibody-based immunotherapeutic strategies. Collectively, our findings suggest that the therapeutic potential of Vγ9Vδ2 T cells may depend not only on the magnitude of γδ T cell expansion but also on the qualitative composition of distinct functional subsets within the Vγ9Vδ2 T cell compartment.

## Data Availability

The sequencing data have been deposited in the Gene Expression Omnibus (GEO) under accession number GSE328705. Any additional data are available from the corresponding authors upon reasonable request. Publicly available data re-analyzed in this study are available from GEO under accession GSE223809.
