# Supplementary material for: Divergent cytotoxic and inflammatory functions of intratumoral Vδ2+ γδ T cells in renal cell carcinoma
Source: Front Immunol. 2026 Jul 17;17:1864165. doi: 10.3389/fimmu.2026.1864165 (PMC13423854; doi:10.3389/fimmu.2026.1864165)
Supplement: Supplementary file 1 [file Image1.pdf]

Supplementary Fiugre 1

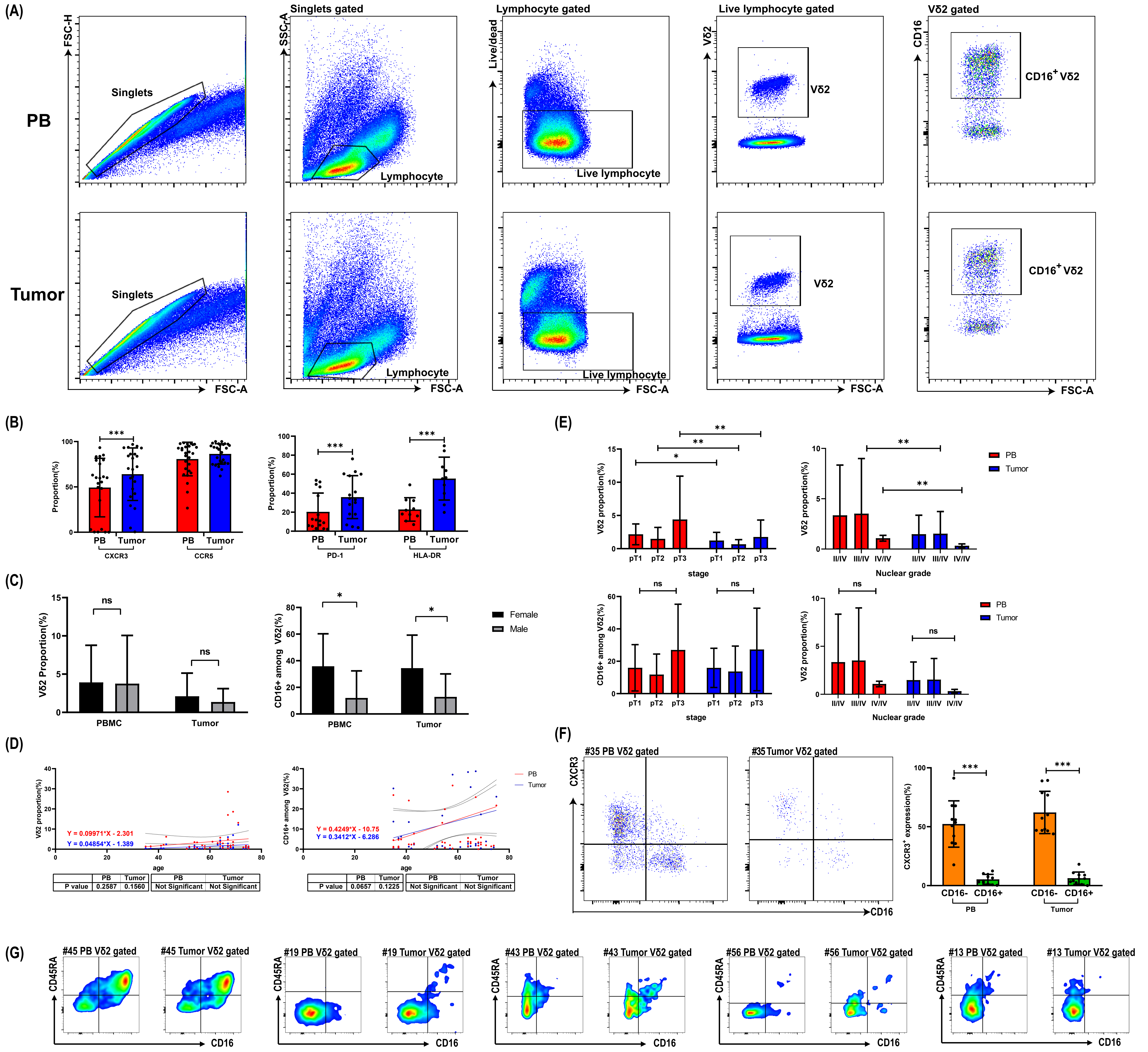

Supplementary Figure 1. Patient demographics and phenotypic characterization of V$\delta 2^+$ $\gamma\delta$ T cells from PBMC and tumor.

A Representative gating strategy for V$\delta 2^+$ $\gamma\delta$ T cells in PB (top) and tumor (bottom): singlets → lymphocytes → live lymphocytes → V$\delta 2^+$ cells → $CD16^+$ and $CD16^-$ V$\delta 2^+$ subsets.
B Comparison of activation and chemokine receptor expression on V$\delta 2^+$ T cells between PBMC (red) and tumor (blue). Proportions of $CXCR3^+$, $CCR5^+$ (left), PD-1^+, and HLA-DR^+ (right) V$\delta 2^+$ T cells are shown.
C V$\delta 2^+$ T cell proportion (left) and CD16 expression on V$\delta 2^+$ T cells (right) compared between female and male patients in PBMC and tumor. ns, not significant.
D Correlation between patient age and V$\delta 2^+$ T cell proportion or CD16 expression on V$\delta 2^+$ T cells in PBMC and tumor. Linear regression lines are shown; correlation coefficients (r) and p-values were determined by Pearson correlation.
E V$\delta 2^+$ T cell proportion (top) and CD16 expression on V$\delta 2^+$ T cells (bottom) according to clinical T stage (pT1–pT3) and nuclear grade in PB (red) and tumor (blue).
F Representative pseudocolor dot plots of CXCR3 versus CD16 on V$\delta 2^+$ T cells from PB and tumor with the analysis gates indicated (left). Quantification of CXCR3 expression in $CD16^-$ (orange) and $CD16^+$ (green) V$\delta 2^+$ T cell subsets from PBMC and tumor (right).
G Per-patient representative smoothed pseudocolor dot plots of CD45RA versus CD16 on PB and tumor V$\delta 2^+$ T cells.
Detailed patient information is provided in Supplementary Table 1. Data are shown as mean ± SD. Statistical significance was determined by paired t-test for the upper row of D (V$\delta 2^+$ T cell proportion) and for panels E and F, and by unpaired t-test (or Mann–Whitney U test) for panel B and the lower row of D; correlations in C were assessed by Pearson correlation. ns, not significant; *$p < 0.05$, **$p < 0.01$, ***$p < 0.001$.
