# Supplementary material for: Divergent cytotoxic and inflammatory functions of intratumoral Vδ2+ γδ T cells in renal cell carcinoma
Source: Front Immunol. 2026 Jul 17;17:1864165. doi: 10.3389/fimmu.2026.1864165 (PMC13423854; doi:10.3389/fimmu.2026.1864165)
Supplement: Supplementary file 2 [file Image2.pdf]

Supplementary Fiugre 2

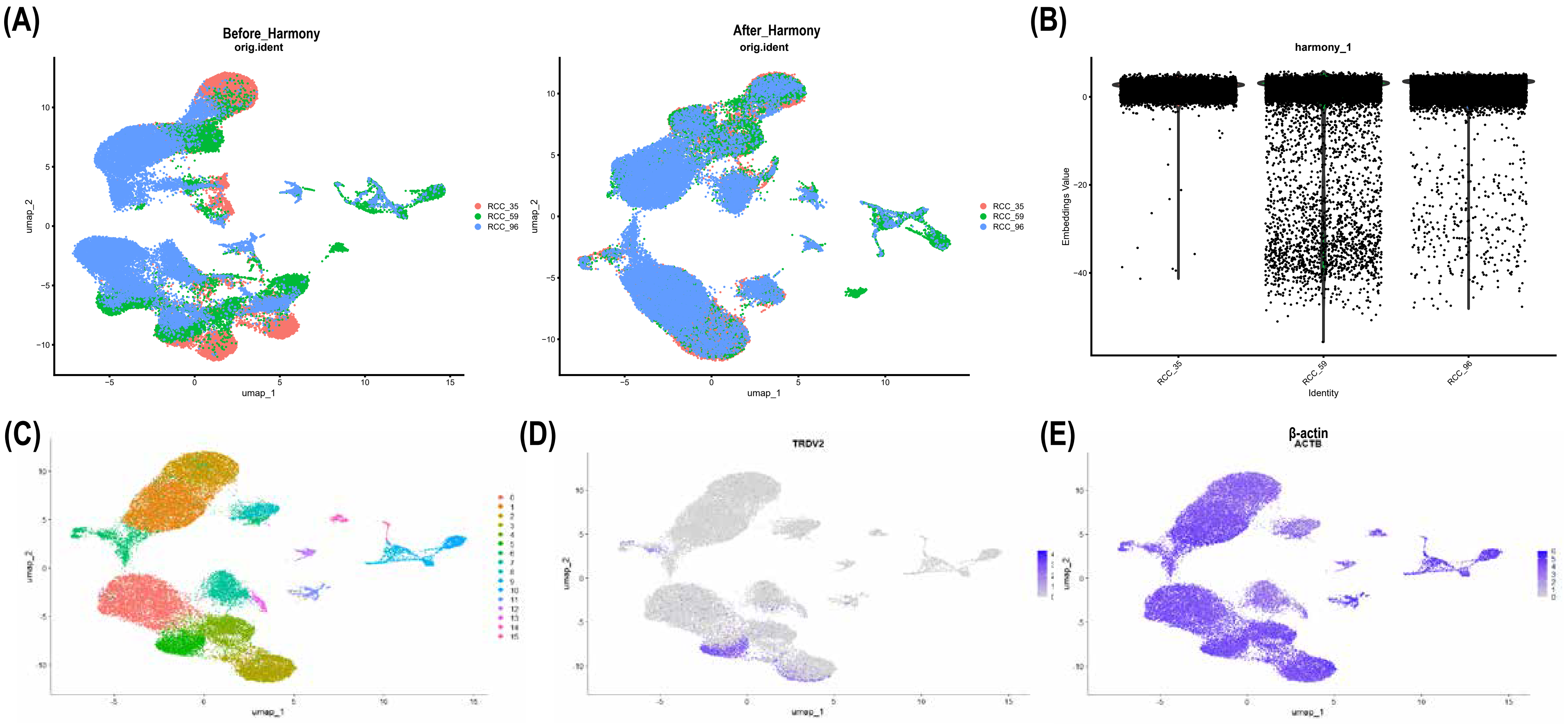

Supplementary Figure 2. Quality Control, Identity Validation, and Batch Effect Correction of Single-cell Data

A-B Harmony integration of three patients' scRNA-seq data across technical batches

A UMAP embeddings of the global single-cell dataset from γδ T and B cells of three patients (#1, #2, and #3) before (left) and after (right) Harmony integration.
The "Before Harmony" plot displays significant batch effects and inter-individual variability, with cells forming distinct, patient-specific islands.
After processing with Harmony, the post-integration UMAP demonstrates homogeneous mixing and overlapping of cells from all three samples,
indicating that batch effects are successfully mitigated and cells can be grouped by shared biological states rather than patient identities.

B Violin plot showing the distribution of the Harmony embedding (indicated as harmony) across the three patient samples.
Here, Harmony embedding values represent the positions of individual cells along a batch-corrected low-dimensional axis learned by the Harmony algorithm.
The nearly identical distributions of harmony values among #1, #2, and #3 samples indicate that this major axis of variation is shared across patients and is no longer driven by patient-specific technical biases,
confirming that cells from different individuals have been successfully aligned into a common integrated space.

C-E UMAP feature plots showing all defined clusters **(C)**, alongside the expression distribution of the Vδ2 T cell receptor gene TRDV2 **(D)** and the housekeeping gene ACTB (β-actin) **(E)**.
