# Supplementary material for: Divergent cytotoxic and inflammatory functions of intratumoral Vδ2+ γδ T cells in renal cell carcinoma
Source: Front Immunol. 2026 Jul 17;17:1864165. doi: 10.3389/fimmu.2026.1864165 (PMC13423854; doi:10.3389/fimmu.2026.1864165)
Supplement: Supplementary file 3 [file Image3.pdf]

Supplementary Figure 3

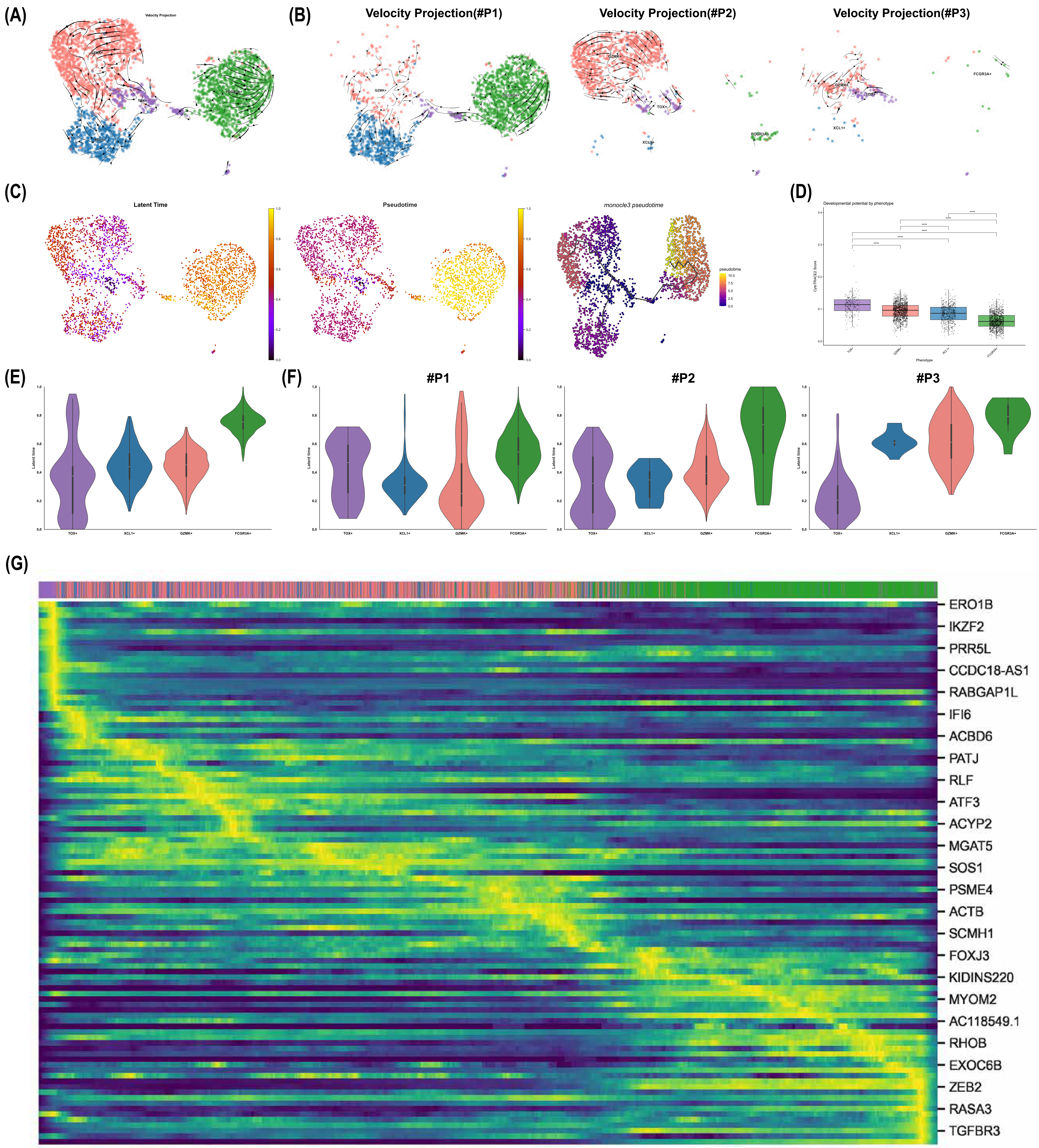

Supplementary Figure 3. Transcriptional Dynamics and Developmental Potential of Vδ2+ T Cells.

A RNA velocity vector field visualized as streamlines on the UMAP embedding, indicating the predicted directional flow of differentiation based on splicing kinetics.
B Per-sample RNA velocity projections shown separately for each patient (#P1–#P3), demonstrating that the velocity field is reproducible across individual donors.
C Feature plots of developmental timelines projected onto the Seurat UMAP space. The panels display (from left to right): latent time derived from the scVelo dynamical model, scVelo-derived velocity pseudotime, and single-cell trajectory pseudotime inferred by Monocle3
D Boxplot comparing the developmental potential across the four clusters, quantified by CytoTRACE 2 potency scores. Statistical significance was determined to highlight the highest developmental potential in the TOX+ cluster (****$p < 0.0001$)..
E Violin plot depicting the distribution of scVelo-derived latent time across the identified Vδ2+ T cell clusters.
F Per-patient (#P1, #P2, #P3) violin plots of latent time across clusters, confirming that the ordering is preserved in each individual donor.
G Heatmap displaying the expression dynamics of selected genes along scVelo-derived latent time across Vδ2^{+} T clusters.
