# Supplementary material for: Divergent cytotoxic and inflammatory functions of intratumoral Vδ2+ γδ T cells in renal cell carcinoma
Source: Front Immunol. 2026 Jul 17;17:1864165. doi: 10.3389/fimmu.2026.1864165 (PMC13423854; doi:10.3389/fimmu.2026.1864165)
Supplement: Supplementary file 4 [file Image4.pdf]

# Supplementary Figure 4

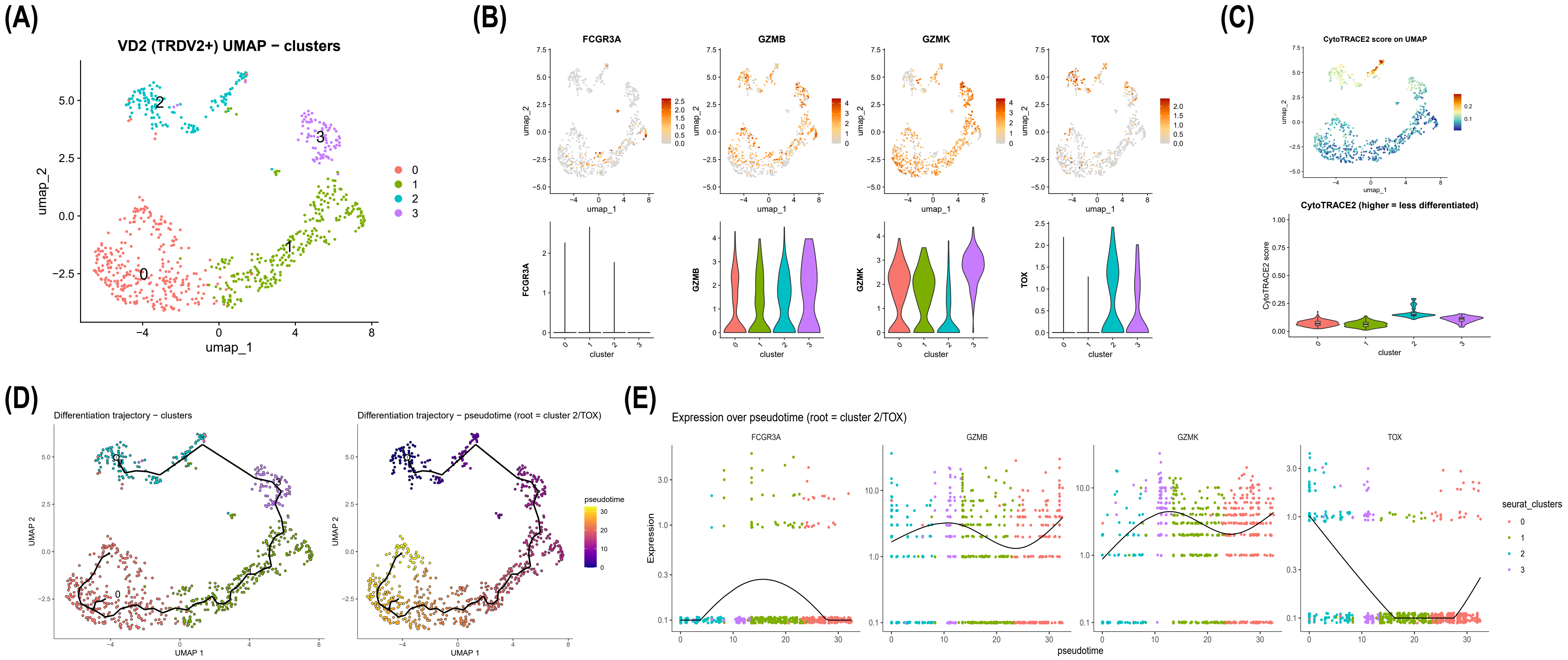


**Supplementary Figure 4. Differentiation trajectory of intratumoral Vδ2$^{+}$ γδ T cells in a public RCC scRNA-seq dataset. Re-analysis of TRDV2$^{+}$ (Vδ2$^{+}$) γδ T cells (n = 788 cells) gated from the public renal cell carcinoma γδ T-cell scRNA-seq dataset GSE223809.**

**A** UMAP embedding of Vδ2$^{+}$ cells colored by unsupervised cluster (clusters 0–3).
**B** Expression of FCGR3A (CD16), GZMB, GZMK and TOX shown as UMAP feature plots (top) and as per-cluster violin plots (bottom).
**C** Differentiation potential estimated by CytoTRACE2, shown on the UMAP (top) and as per-cluster violin plots (bottom); higher CytoTRACE2 score indicates a less differentiated state. Cluster 2 shows the highest score, identifying it as the least differentiated (most stem-like) population.
**D** Monocle3 differentiation trajectory of Vδ2$^{+}$ cells colored by cluster (left) and by pseudotime (right). Pseudotime root was set at cluster 2.
**E** Expression of FCGR3A, GZMB, GZMK and TOX along pseudotime (root = cluster 2/TOX). TOX is highest at the root and declines with pseudotime, whereas the cytotoxic effector genes GZMK, GZMB and CD16 (FCGR3A) are up-regulated relative to the root as differentiation proceeds.
