# Supplementary material for: Divergent cytotoxic and inflammatory functions of intratumoral Vδ2+ γδ T cells in renal cell carcinoma
Source: Front Immunol. 2026 Jul 17;17:1864165. doi: 10.3389/fimmu.2026.1864165 (PMC13423854; doi:10.3389/fimmu.2026.1864165)
Supplement: Supplementary file 5 [file Image5.pdf]

Supplementary Figure 5

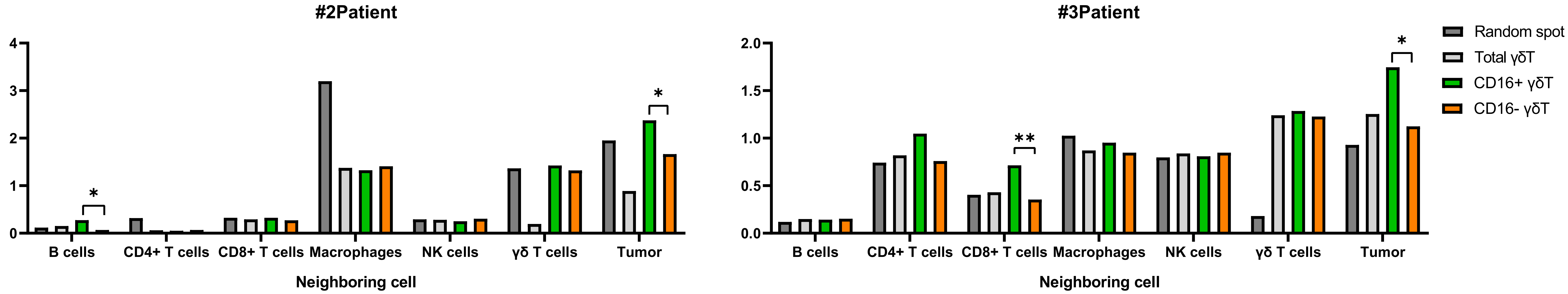

Supplementary Figure 5. Neighborhood composition analysis of CD16+ and CD16- γδ T cell subsets in spatial transcriptomics.

Bar graphs show average numbers of indicated cells neighboring to CD16- γδ T cells (orange), CD16+ γδ T cells (green), random spots (gray), and total γδ T cells (light gray) in patient #2 (left) and patient #3 (right). Y-axis represents mean number of neighboring spots for each cell type category (B cells, CD4+ T cells, CD8+ T cells, macrophages, NK cells, γδ T cells, tumor).
