# Supplementary material for: Divergent cytotoxic and inflammatory functions of intratumoral Vδ2+ γδ T cells in renal cell carcinoma
Source: Front Immunol. 2026 Jul 17;17:1864165. doi: 10.3389/fimmu.2026.1864165 (PMC13423854; doi:10.3389/fimmu.2026.1864165)
Supplement: Supplementary file 6 [file Image6.pdf]

Supplementary Figure 6

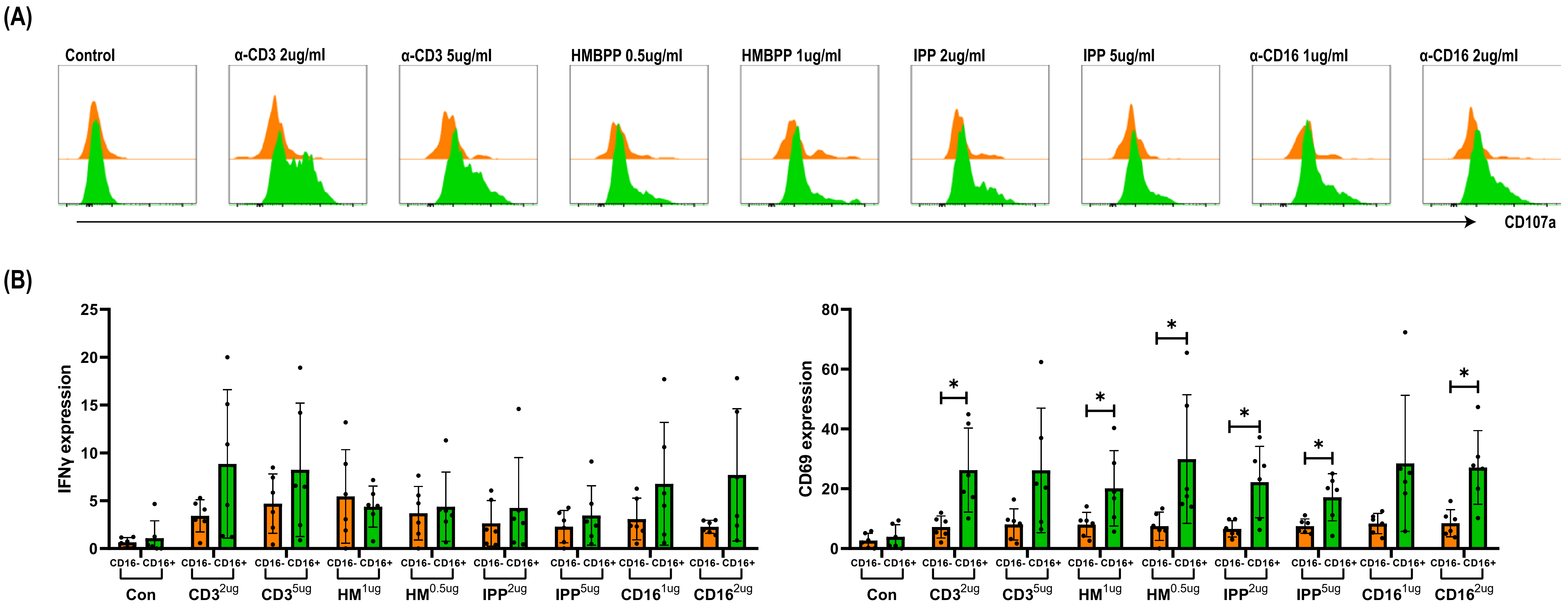

Supplementary Figure 6. Expression of CD107a, IFNγ and CD69 in $CD16^-$ and $CD16^+$ $V\delta2^+$ γδ T cells from healthy donors that are untreated or treated with given stimuli.

A Representative flow cytometry histograms of surface CD107a in $CD16^-$ (orange) and $CD16^+$ (green) $V\delta2^+$ γδ T cells following 4-hour treatment with the indicated stimuli.
B Levels of intracytoplasmic IFNγ (left) or surface CD69 (right) were shown in $CD16^-$ (orange) and $CD16^+$ (green) $V\delta2^+$ γδ T cells following 4-hour treatment with various stimuli: control medium (Con), anti-CD3 antibody (2 or 5 μg/ml), HMBPP (0.5 or 1 μg/ml), IPP (2 or 5 μg/ml), and anti-CD16 antibody (1 or 2 μg/ml).
Data are shown as mean ± SD; statistical significance was determined by paired t-test for matched $CD16^-$ and $CD16^+$ $V\delta2^+$ γδ T cell subsets from the same donor. *$p < 0.05$, **$p < 0.01$, *$p < 0.001$.
