# Supplementary material for: Divergent cytotoxic and inflammatory functions of intratumoral Vδ2+ γδ T cells in renal cell carcinoma
Source: Front Immunol. 2026 Jul 17;17:1864165. doi: 10.3389/fimmu.2026.1864165 (PMC13423854; doi:10.3389/fimmu.2026.1864165)
Supplement: Supplementary file 7 [file Table1.pdf]

Supplementary Table 1

Patient no	Sex	Age	Stage	Tumor size	Nuclear grade
1	F	42	pT2bN0	13.5x12.5 cm	III/IV
2	F	64	pT1b	4.1x3 cm	II/IV
3	M	74	pT2a	7.3x6.0	III/IV
4	M	56	pT3	8.6x7.5 cm	III/IV
5	F	64	pT1b	4.1x3.1 cm	III/IV
6	M	69	pT3a	6.3x6 cm	III/IV
7	M	57	pT3a	6.5x5 cm	III/IV
8	M	61	pT3a	8.5x7 cm	III/IV
9	F	65	pT1b	5.5x5.0 cm	III/IV
10	M	55	pT3a	6x5 cm	III/IV
11	M	67	pT3a N0	6.5x4.3 cm	II/IV
12	M	51	pT3a Nx	8x7 cm	III/IV
13	M	54	pT3a	5.3x4.5x3.5 cm	II/IV
14	F	68	pT3a N0	11.5x9 cm	III/IV
15	F	75	pT3a Nx	6.8x5.6 cm	III/IV
16	M	69	pT1b	5x4.5 cm	III/IV
17	M	67	pT1b	4.2x3.5 cm	III/IV
18	F	67	pT3a	6.7x5.5 cm	III/IV
19	F	37	pT3a Nx	9x6 cm	IV/IV
20	M	69	pT2a Nx	7.8x5 cm	III/IV
21	F	60	pT2a	7.5x6 cm	II/IV
22	M	53	pT2a	8x7.5 cm	III/IV
23	F	63	pT1b	5.2x2.7 cm	III/IV
24	M	42	pT1a	2.2x1.5x1.3 cm	II/IV
25	F	64	pT3a N0	7.5x3.5x2 cm	III/IV
26	M	55	pT3a N0	10.5x6 cm	III/IV
27	M	35	pT2a N0	7.2x5 cm	III/IV
28	F	65	pT1b Nx	6.3x5 cm	III/IV
29	M	78	pT2a Nx	8x5 cm	III/IV
30	M	43	pT2 Nx M1	7.3x5 cm	III/IV
31	M	41	pT3a NX	9.5x6.5 cm	II/IV
32	M	75	pT3a Nx	7.3x6.5 cm	III/IV
33	F	68	pT3a	7.6x5.5 cm	III/IV
34	M	58	pT2a	7.4x6.3 cm	III/IV
35	F	71	pT3a	7.4x5.5 cm	II/IV
36	M	67	pT3a Nx	6x5 cm	III/IV
37	M	70	pT3a	7.6x5.3 cm	III/IV
38	M	54	pT1b	6.3x5.0 cm	IV/IV
39	M	44	pT3a	9.4x7 cm	III/IV
40	M	63	pT3a	4.6x3 cm	III/IV
41	M	75	pT1b	5x4.6 cm	III/IV
42	M	68	pT3a NX	8.3x5 cm	IV/IV
43	M	63	pT3a N0	9.2x8 cm	III/IV
44	F	67	pT3a	2.5x2.5 cm	III/IV
45	F	58	pT1b	5.6x4.5 cm	III/IV
46	M	52	pT3a	10.7x8.5 cm	III/IV
47	M	61	pT2b	10.3x7.3 cm	III/IV
48	M	68	pT2b	10.3x9.2 cm	III/IV
49	M	73	pT3a	8.5x6x6 cm	III/IV
50	M	69	pT3a Nx	7.2x6 cm	II/IV
51	F	71	pT3a N0	6.4x4 cm	III/IV
52	F	50	pT1b	6.3x4.7 cm	II/IV
53	M	31	pT1b N0	6.2x5.5 cm	III/IV
54	M	52	pT3a N0	3.8x2.5 cm	II/IV
55	M	60	pT1b	5.1x3.5 cm	III/IV
56	F	45	pT1b	5.4x4.2 cm	III/IV
57	M	67	pT2a Nx	7.2x3.8 cm	III/IV
58	M	74	pT3a Nx	7.1x6 cm	III/IV
59	F	38	pT2a Nx	7.2x6.1 cm	III/IV
60	M	66	pT3a Nx	5.1x3.5 cm	III/IV
61	F	72	pT3a Nx	5.5x4 cm	III/IV
62	F	39	pT2a	8x6 cm	III/IV
63	M	74	pT3 NX	10x7 cm	II/IV
64	F	58	pT3a Nx	8.5x7 cm	III/IV
65	M	68	pT1a	3.7x3 cm	III/IV
66	M	38	pT3a Nx	5.7 x4 cm	III/IV
67	M	58	pT2b	13 x10 cm	II/IV
**NGS #P1**	**M**	**67**	**pT3a Nx**	**11x7 cm**	**III/IV**
**NGS #P2**	**M**	**52**	**pT3a**	**5.8x5 cm**	**III/IV**
**NGS #P3**	**M**	**47**	**pT1a Nx**	**3x3 cm**	**III/IV**
