# Supplementary material for: Divergent cytotoxic and inflammatory functions of intratumoral Vδ2+ γδ T cells in renal cell carcinoma
Source: Front Immunol. 2026 Jul 17;17:1864165. doi: 10.3389/fimmu.2026.1864165 (PMC13423854; doi:10.3389/fimmu.2026.1864165)
Supplement: Supplementary file 8 [file Table2.pdf]

**Supplementary Table 2**

| Target | Clone | Fluorochrome | Vendor | Cat# |
|---|---|---|---|---|
| TCR Vδ2 | B6 | BV510 | BioLegend | 331432 |
| HLA-DR | L243 | BV421 | BioLegend | 307636 |
| CCR5 | J418F1 | BV421 | BioLegend | 359118 |
| CCR5 | J418F1 | FITC | BioLegend | 359120 |
| CD3 | OKT3 | BV421 | BioLegend | 317344 |
| CD107a | H4A3 | PerCP-Cy5.5 | BioLegend | 328616 |
| CD69 | FN50 | PerCP-Cy5.5 | BioLegend | 310926 |
| CD69 | FN50 | PE-Cy7 | BioLegend | 310912 |
| CD45RA | HI100 | PerCP-Cy5.5 | BioLegend | 304122 |
| CD45RA | HI100 | PE-Cy7 | BioLegend | 304126 |
| CD16 | 3G8 | PerCP-Cy5.5 | eBioscience | 46-0166-42 |
| CD16 | 3G8 | PE-Cy7 | BioLegend | 302016 |
| CD16 | B73.1 | APC | BioLegend | 360706 |
| CD103 | BER-ACT8 | APC | BioLegend | 350216 |
| PD-1 | A17188B | PE-Cy7 | BioLegend | 621616 |
| GZMB | QA16A02 | PE-Cy7 | BioLegend | 372214 |
| CD28 | CD28.2 | PE-Cy7 | BioLegend | 302926 |
| CD28 | CD28.2 | PE | BioLegend | 302940 |
| NKG2A | S19004C | PE-Cy7 | BioLegend | 375114 |
| NKG2A | S19004C | PE | BioLegend | 375104 |
| GZMK | GM26E7 | PE | BioLegend | 370512 |
| CXCR3 | G025H7 | PE | BioLegend | 353706 |
| CX3CR1 | 2A9-1 | PE-Cy7 | BioLegend | 341612 |
| CD27 | O323 | PE | eBioscience | 12-0279-42 |
| CD27 | O323 | FITC | BioLegend | 302806 |
| TCR Vδ1 | TSA8.20 | FITC | Invitrogen | TCR2730 |
| TCRϒδ | B1 | PerCP-Cy5.5 | BioLegend | 331224 |
| TCRϒδ | B1 | PE-Cy7 | BioLegend | 331222 |
| Viability | - | eFluor 780 | eBioscience | 65-0865-14 |
